# MoBC-Net: computing network inter-module distance and module-based betweenness centrality

**DOI:** 10.1093/bioadv/vbag279

**Published:** 2026-10-03

**Authors:** Hyekyoung Lee, Hanbi Lee, Yoomi Baek, Ahyoung Choi, Hyunjin Shin

**Affiliations:** MOGAM Institute for Biomedical Research, Seoul 06730, Republic of Korea; MOGAM Institute for Biomedical Research, Seoul 06730, Republic of Korea; MOGAM Institute for Biomedical Research, Seoul 06730, Republic of Korea; MOGAM Institute for Biomedical Research, Seoul 06730, Republic of Korea; MOGAM Institute for Biomedical Research, Seoul 06730, Republic of Korea

## Abstract

**Motivation:**

Analyzing disease-associated modules within gene co-expression and protein-protein interaction networks can improve disease-mechanism interpretation from multi-omics data. Disease-relevant interactions may also extend across module boundaries through genes not identified as differentially expressed, requiring consideration of both local module structure and the broader network context. Global centrality measures may preferentially rank highly connected hubs without distinguishing module-pair-specific connectors, while common size- and degree-matched random-module schemes do not explicitly account for local module compactness.

**Results:**

To address these challenges, we introduce MoBC‑Net, a multi‑scale network analysis framework that quantifies interactions among disease-associated modules and prioritizes candidate link genes. MoBC-Net selects interacting module pairs using a closest-distance measure, and identifies link genes via module betweenness centrality. Statistical significance is assessed using an adaptive binning and randomization scheme that preserves module size and node degree, while favoring local compactness. When applying MoBC-Net to TCGA breast cancer and cancer cachexia data, inter-module distances showed data-dependent concordance with those computed via gene ontology semantic similarity. In addition, link genes significantly overlapped in pathways commonly enriched by the module pair. Our case studies highlighted EPHA2 linking PI3K‑Akt modules in breast cancer and Gnai2 linking Apelin and cAMP signaling pathways affecting muscle hypertrophy and regeneration in cachexia. We believe that MoBC-Net’s multi-scale approach, which progressively infers disease gene modules and key link genes, can improve network-guided investigation of disease mechanisms and therapeutic hypothesis generation.

**Availability and implementation:**

An open-source R implementation of the MoBC-Net is available at GitHub (https://github.com/mogam-ai/MoBC-Net).

## 1 Introduction

Understanding complex disease mechanisms benefits from a systems-level view of molecular changes and their relationships. Systems-level analyses of gene expression profiles are widely used to identify modules of genes with coordinated expression patterns and biological processes associated with disease (Allayee *et al.* 2023). Differentially expressed genes (DEGs) are commonly mapped onto protein-protein interaction (PPI) networks to identify disease-associated subnetworks and prioritize highly connected hub genes (Peng *et al.* 2017, Wu and Xi 2021). Complementing these node- and subnetwork-level analyses, metric-backbone approaches use global shortest-path structure to remove redundant edges while preserving shortest paths and much of the original community organization (Brattig Correia *et al.* 2023, Brattig-Correia *et al.* 2024).

In network medicine, a *disease gene module* refers to a localized group of disease-associated genes or proteins within an interaction network that participate in related biological processes (Menche *et al.* 2015). However, disease modules are not necessarily functionally homogeneous and may consist of multiple distinct subnetworks. Characterizing the genes and paths that connect these subnetworks is important because it can reveal relationships among disease-associated processes that may not be captured by analyses confined to individual subnetworks (Guney *et al.* 2016, Garcia-Vaquero *et al.* 2018). Previous studies have therefore extended network analysis beyond individual modules by quantifying inter-module relationships. These approaches have been used to characterize disease-disease relationships and to assess the network proximity between drug targets and disease gene modules for repurposing (Barabási *et al.* 2011, Menche *et al.* 2015, Guney *et al.* 2016). Cross-disease analyses have focused on connector genes linking distinct disease gene modules, whereas drug repurposing studies have focused on targets that are topologically close to, or strongly connected with, disease-associated gene sets (Guney *et al.* 2016, Tao *et al.* 2020, Guala and Sonnhammer 2022, Chen *et al.* 2024, Gross *et al.* 2025, Pour *et al.* 2025).

CytoHubba and CentiScaPe provide node-level centrality analyses for identifying key nodes and subnetworks in biological networks (Scardoni *et al.* 2009, Chin *et al.* 2014). Global node-level centralities summarize node importance across the entire network, whereas specific betweenness (S2B) and flow centrality score (FCS) evaluate betweenness-like importance with respect to specified module pairs (Garcia-Vaquero *et al.* 2018, Maiorino *et al.* 2020). These module-pair-specific measures can identify connector genes that may not be highly ranked by degree or conventional betweenness centrality. At the module-pair level, proximity methods quantify shortest-path separation, whereas crosstalk methods assess whether the observed interaction between gene sets differs from a null expectation (Menche *et al.* 2015, Guney *et al.* 2016, Guala and Sonnhammer 2022). Methods such as NEAT, BinoX, and ANUBIX provide statistical tests of gene-set crosstalk using different null models (Menche *et al.* 2015, Guney *et al.* 2016, Guala and Sonnhammer 2022). A remaining challenge is to evaluate both module-pair proximity and connector-gene importance within a common, topology-aware framework that is practical for repeated randomization.

Evaluating the statistical significance of module distance and connector-gene scores is critical for prioritizing network findings. Existing studies commonly compare observed values with null distributions generated through edge rewiring or degree-matched random-set sampling (Guney *et al.* 2016, Garcia-Vaquero *et al.* 2018, Maiorino *et al.* 2020). Degree-preserving edge rewiring retains node degrees but alters local connectivity and must be repeated for each null replicate. Degree-matched random-set sampling is more direct, but it does not explicitly constrain the sampled genes to form locally cohesive modules. For example, Güney *et al.* used degree bins to construct degree-matched random gene sets and thereby control degree-related bias, but these random sets were not required to form coherent modules (Guney *et al.* 2016).

S2B assesses score specificity using randomized seed sets and degree-preserving rewired networks. FCS generates degree‑matched random seed sets and expands them using the DIseAse MOdule Detection (DIAMOnD) algorithm, thereby applying the same module-construction procedure to the observed and null modules (Garcia-Vaquero *et al.* 2018, Maiorino *et al.* 2020). DIAMOnD recalculates the connectivity significance of candidate nodes at each expansion step and adds the most significant candidate (Ghiassian *et al.* 2015). When used for repeated null-model generation, this rescoring must be repeated for every randomized seed set and expansion step.

DIAMOnD is a seed-expansion method rather than a community-detection algorithm that partitions the entire input network. Separately, the granularity of modules produced by community-detection algorithms is important for downstream inter-module analysis: coarse partitions may merge functionally distinct subnetworks, whereas overly fine partitions may fragment biologically coherent modules (Fortunato and Barthélemy 2007, Traag *et al.* 2019).

In this study, we introduce MoBC-Net, a unified multi-scale framework that quantifies inter-module distance and identifies key link genes connecting disease-associated modules (Fig. 1). At the local scale, MoBC-Net identifies modules within the PPI subnetwork induced by DEGs, hereafter referred to as the *DEG-overlap subnetwork*. At the global scale, it evaluates the distances and shortest-path interfaces between these modules within the broader PPI network, hereafter referred to as the *background network*. This design allows genes outside the two focal modules, including genes not identified as differentially expressed, to be considered as candidate connectors. We use closest distance to identify statistically close module pairs. Module betweenness centrality (MoBC) is used to prioritize genes contributing to the shortest paths connecting each pair (Guney *et al.* 2016). To assess statistical significance, we introduce adaptive binning (ABin) and a random module generation scheme that matches module size and node degree distribution while favoring local compactness (RandSDM). ABin defines degree bins dynamically for each module, whereas RandSDM preferentially samples candidate nodes that are topologically close to the already sampled nodes. This combination provides topology-aware null models for standardizing inter‑module distances and centrality scores.

**Figure 1:**
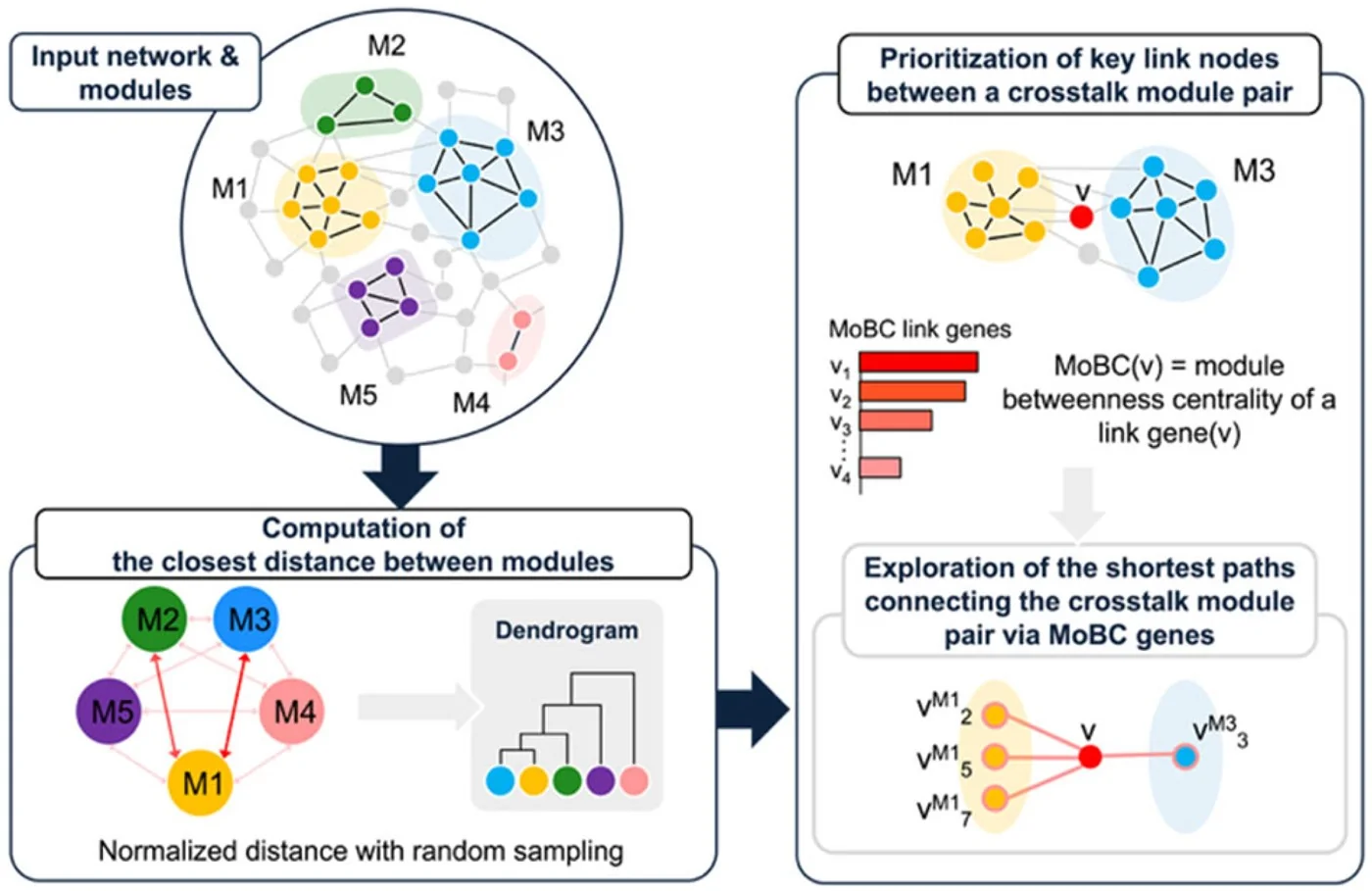
MoBC-Net: a multi-scale network framework for disease gene identification.

We demonstrate the utility of MoBC-Net through case studies using The Cancer Genome Atlas Breast Invasive Carcinoma (TCGA-BRCA) and mouse cancer cachexia (CAC) data. In both datasets, the DEG-overlap subnetworks were partitioned into several modules. By characterizing genes within and between disease-associated modules, MoBC-Net adds an inter-module perspective to conventional DEG and pathway analyses and prioritizes connector candidates for further investigation and future experimental validation. Together, these case studies illustrate how MoBC-Net can translate modular network structure into interpretable hypotheses about disease mechanisms.

## 2 Materials and methods

### 2.1 Data preprocessing

#### 2.1.1 PPI network construction and module identification

PPI networks for both human and mouse data were constructed using the STRING v12.0 database with a confidence threshold of 0.7 (Szklarczyk *et al.* 2023). For the TCGA‑BRCA dataset, we constructed a human PPI network comprising 15 867 proteins and 235 783 interactions by filtering genes based on standard deviation of transcripts per million (TPM) values greater than 0.1. For the CAC dataset, a mouse PPI network comprising 12 543 proteins and 169 320 interactions was constructed by using genes with non-zero expression counts.

#### 2.1.2  Identification of DEG-associated modules

TCGA‑BRCA expression counts (224 paired tumour/normal samples) were downloaded from the UCSC Xena portal (Love *et al.* 2014, Goldman *et al.* 2020). Differential expression analysis was performed with DESeq2 (v1.24.1) (Love *et al.* 2014), identifying 1635 genes with $|\log_{2}\mathrm{Fold}\;\mathrm{Change}\;(FC)|>2$ and Benjamini-Hochberg (BH)-adjusted P<0.01 (Supplementary Material Fig. S1, available at supplementary material  *Bioinformatics Advances* online).

Mouse RNA‑seq data (GEO GSE157251) were quality-checked with FastQC (v0.11.9), trimmed with TrimGalore (v0.6.6), aligned to the GRCm38 reference genome using STAR (v2.7.9a), and quantified by RSEM (v1.3.3) (Andrews 2010, Li and Dewey 2011, Martin 2011, Dobin *et al.* 2013, Clapes *et al.* 2021, Zhong *et al.* 2022). DEGs between cachectic and control groups were again assessed with DESeq2, identifying 1211 genes at $|\log_{2}FC|>1$ and BH-adjusted P<0.05 (Supplementary Material Fig. S1, available at supplementary material  *Bioinformatics Advances* online). Further details on the rationale for the dataset-specific DEG thresholds and robustness to nearby threshold choices are provided in Supplementary Material Sec. 2A, available at supplementary material  *Bioinformatics Advances* online.

A DEG-overlap subnetwork is the subgraph induced by mapped DEG nodes and edges connecting them in the corresponding background network. Community detection was performed on the DEG-overlap subnetwork, whereas inter-module distances, randomization, and link-gene analyses were performed using the background network.

For the primary MoBC-Net analysis, modules were detected using Walktrap algorithm (Pons and Latapy 2005). In TCGA-BRCA, Walktrap was run with four random-walk steps, and eight modules containing more than 20 genes were retained. In the cancer cachexia network, Walktrap was run with three random-walk steps, and nine modules containing more than 10 genes were retained.

To assess sensitivity to the community-detection algorithm and its effective resolution, we compared Walktrap, Leiden, Louvain, and Infomap across BRCA-PPI, CAC-PPI, and a BRCA weighted gene co-expression network (BRCA-WGCN) (Supplementary Material Sec. 2B, available at supplementary material  *Bioinformatics Advances* online). Walktrap was evaluated using 3–9 random-walk steps, Leiden and Louvain using resolution values from 0.6 to 4.0, and Infomap using Markov-time values from 0.25 to 4.0. The comparison was based on six evaluation factors: stability, reproducibility, size appropriateness, graph quality, enrichment consistency, and background-network cohesion. Parameters were selected before any downstream MoBC, S2B, or FCS result was examined. Detailed factor definitions, parameter grids, weighting sensitivity analyses, and selected configurations are provided in Supplementary Material Sec. 3, available at supplementary material  *Bioinformatics Advances* online.

The complete downstream analysis was then applied to the selected configuration of each community-detection method. Inter-module distance analysis was additionally performed across all analyzable parameter settings to assess resolution sensitivity. Functional-enrichment consistency and biological annotation of the selected modules were evaluated using Gene Ontology (GO) and Kyoto Encyclopedia of Genes and Genomes (KEGG) pathway (Ashburner *et al.* 2000, Kanehisa and Goto 2000, Yu *et al.* 2012, Wu *et al.* 2021).

#### 2.1.3 Assumptions and scope of MoBC-Net

MoBC-Net takes as input an undirected background network and a non-overlapping set of modules whose nodes are contained within the network. Its multi-scale design treats the input modules as local units and evaluates their pairwise proximity and candidate link genes in the broader background network. In this study, unweighted shortest-path distances were computed within the giant connected component of each background network. The framework is not intrinsically tied to Walktrap. Modules can be obtained using alternative community-detection algorithms or other biologically justified procedures, provided that the resulting module sets are non-overlapping. The input modules need not cover all nodes in the background network, allowing nodes outside the two focal modules to be evaluated as candidate link genes. The network-specific module-size filters and the requirement of at least five retained modules were study-specific feasibility criteria rather than intrinsic requirements of MoBC-Net.

Because this multi-scale analysis depends on both the input partition and the background network, its results may vary with the community-detection method and resolution. Its informativeness may also depend on the topology and density of the background network. MoBC-Net may be most informative in relatively sparse gene interaction networks, such as PPI networks. In denser networks with many short paths and more consolidated modules, the number of separable intermodule relationships available for evaluation may be reduced. Shortest paths are used as topological proxies for potential connectivity and should not be interpreted as direct biochemical or causal routes. Although DEG selection introduces condition-specific information, the background network is treated as static and does not model temporal, cell-type-specific, or sample-specific changes in molecular interactions.

### 2.2 Inter-module distance and module betweenness centrality

Given background network and disease gene modules, MoBC-Net quantifies the interaction between any two modules in a network by adopting the closest inter-module distance metric (Guney *et al.* 2016) and evaluates each gene’s contribution to inter-module connectivity by inducing MoBC from the closest distance.

Suppose a background network G=(V,E) is given, where V is the set of nodes and E is the set of edges. The shortest path length between nodes, u, w∈V is defined as the minimum number of edges connecting them, and is denoted by $d_{uw}$. For two given modules U and W⊂V in G, we define the set of closest node pairs from each node u∈U to its nearest node w∈W as


$$S_{\mathrm{MoBC}}^{UW}=\{(u,w)\in U\times W|d_{uw}=\underset{w\prime \in W}{\min}d_{uw\prime }\},$$

And analogously, the set of the closest node pairs from W to U as $S_{\mathrm{MoBC}}^{WU}$. The closest distance between the two modules is then defined as the average shortest-path length from each node in one module to its nearest neighbor in the other module (and vice versa) as follows (Guney *et al.* 2016):


$$D_{\mathrm{MoBC}}(U,W)=\frac{1}{\left|U|+|W\right|}(\sum_{u\in U}\underset{w\in W}{\min}d_{uw}+\sum_{w\in W}\underset{u\in U}{\min}d_{wu}),$$

where |U| is the number of nodes in module U. Intuitively, a smaller $D_{\mathrm{MoBC}}(U,W)$ indicates that many nodes in U have direct or very short connections to nodes in W.

During the estimation of the closest distance, the geodesic paths of one-to-many mappings from nodes in U to corresponding nodes in W and vice versa are obtained. We assume that these paths mainly contribute to the connections between U and W, and define MoBC to quantify the contribution of nodes on these paths to connecting between U and W:


$$c_{\mathrm{MoBC}}^{UW}(v)=\frac{1}{\left|S_{\mathrm{MoBC}}^{UW}|+|S_{\mathrm{MoBC}}^{WU}\right|}(\sum_{(u,w)\in S_{\mathrm{MoBC}}^{UW}}\frac{\sigma_{uw}(v)}{\sigma_{uw}}+\sum_{(w,u)\in S_{\mathrm{MoBC}}^{WU}}\frac{\sigma_{wu}(v)}{\sigma_{wu}}),$$

where $\sigma_{uw}$ and $\sigma_{uw}(v)$ are the number of shortest paths between u and w and that through v∈V, respectively. MoBC is bounded in [0,1]. The value of 0 indicates that v is not included in any shortest path between U and W. The value of 1 indicates that it must always pass through v when traveling between U and W.

#### 2.2.1 Comparison of other inter-module betweenness centrality measures

S2B centrality counts how often a node lies on paths between two modules whose length is below a predefined threshold (Garcia-Vaquero *et al.* 2018). For each endpoint pair, S2B records whether a candidate occurs on a qualifying shortest path rather than weighting the candidate by the number of equally short paths for that pair. Consequently, S2B emphasizes genes that recur across multiple short inter-module connections.

FCS treats all shortest paths between two modules as a flow, and measures the fraction of that flow passing through each node (Maiorino *et al.* 2020). It therefore accounts explicitly for shortest-path multiplicity. The original FCS framework uses randomized disease modules to assess whether a candidate’s flow centrality is specific to the observed module pair rather than attributable to general network connectivity.

MoBC also accounts for shortest-path multiplicity but restricts the evaluated endpoint pairs to nearest cross-module pairs. Multiplicity is relevant because several equally short routes may connect the same pair of module-boundary nodes.

In the undirected networks analysed here, S2B and FCS are symmetric because they are evaluated over cross-module endpoint pairs without assigning an intrinsic source–target direction. MoBC first constructs directional closest-pair sets because nearest-neighbour relationships may not be one-to-one, but combines the U→W and W→U sets in the final calculation. Consequently, the final MoBC, S2B, and FCS module-pair measures are all symmetric in the present analysis.

We additionally defined S2B-based and FCS-based inter-module distance measures, as described in Supplementary Material Sec. 4A, available at supplementary material  *Bioinformatics Advances* online. MoBC evaluates shortest paths only for nearest cross-module endpoint pairs, whereas S2B and FCS evaluate a broader set of cross-module endpoint pairs before applying their method-specific selection or weighting rules. This reduces the number of paths that must be enumerated by MoBC, particularly when link-gene scoring is repeated over many randomized module pairs. Formal complexity analyses and empirical runtime comparisons are provided in Supplementary Material Sec. 4B, available at supplementary material  *Bioinformatics Advances* online.

### 2.3 Randomization

MoBC‑Net employs two randomization strategies: (1) ABin, an adaptive binning algorithm that adjusts degree bins based on each module’s degree distribution so that all degree classes are adequately sampled, and (2) RandSDM (Randomization preserving Size, Degree, and Modularity), which generates random modules to normalize inter‑module distances and centralities.

#### 2.3.1 ABin algorithm

ABin constructs degree bins dynamically based on how often nodes of a given degree must be sampled to reproduce the module’s degree distribution. Conceptually, the algorithm proceeds as follows:

**Input**: Network N=(V,E), module sets $\left\{M_{m}\right\}$, number of randomizations $n_{cv}$, threshold coefficient κ (default κ=0.07)

**Output**: Adaptive degree bins $B=\{B_{d}\}$

**1. Initialize.** 

1–1. Construct initial bins $B=\{B_{d}\}$, where each distinct degree d forms its own bin.

1–2. Set a threshold $T=\kappa n_{cv}$.

**2. Estimate sampling demand.** 

For each bin $B_{d}\in B$:

2–1. For each module m, compute

$$p_{d,m}=\frac{\left|B_{d}\cap M_{m}\right|}{\left|B_{d}\right|}.$$

2–2. Compute

$$n_{d}=n_{cv}\underset{m}{\max}p_{d,m}.$$

**3. Merge oversampling-prone bins.** 

Until all bins satisfy $n_{d}<T$,

3–1. Merge $B_{d}$ with an adjacent bin, if $n_{d}>T$.

3–2. Recompute $p_{d,m}$ and $n_{d}$ for the merged bin.

By determining bin boundaries from the module’s degree distribution and the total number of randomizations, ABin ensures that random modules can be drawn fairly across the entire degree spectrum. Unlike fixed-size binning methods, referred to as TBin (Guney *et al.* 2016), ABin automatically expands bins where high‑degree nodes are rare, thereby preventing hub oversampling and producing a more even, modularity‑aware sampling across the network.

#### 2.3.2 RandSDM

RandSDM generates random modules in three steps:

1. *Degree‑matching sequence.* For each module m, determine the degrees of its member nodes and randomly shuffle this sequence. Each entry in the shuffled sequence specifies the degree that the next node in the random module must have, ensuring the final module’s degree distribution matches that of the original.
2. *Sequential distance‑weighted sampling.* For each degree value in the shuffled sequence:
  2-1. Identify the pool of unused candidate nodes with that degree from the ABin‑generated degree bins. If no node has been selected yet, choose one candidate uniformly at random.
  2-2. Otherwise, compute the shortest‐path distance from each candidate in the pool to the nodes already selected. Convert these distances into selection weights so that candidates closer to the existing module have higher probabilities. Sample one candidate according to these weights and remove it from the pool. Append the sampled node to the random module.
3. *Module assembly and repetition.* After iterating through all degree values, assign the resulting set of sampled nodes as a new random module m. Repeat this procedure for each original module. By generating such random modules multiple times (e.g. 1000 per module pair), we obtain a robust null distribution of inter‑module distances and centralities.

We used a Gaussian kernel for the selection weights as follows:


$$\mathrm{selection}\;\mathrm{weight}(v)=\exp\left(-\frac{\underset{u\in U_{t}}{\min}d_{uv}^{2}}{2}\right),$$

where $U_{t}$ was the set of selected nodes during the sequential distance-weighted sampling. This algorithm preserves the original modules’ size and degree sequence while favoring topologically compact selections at each step. Consequently, RandSDM is expected to generate more topologically compact random modules than RandSD, the earlier randomization scheme that matched only module size and degree (Supplementary Material Sec. 5, available at supplementary material  *Bioinformatics Advances* online). The combination of ABin’s balanced degree bins and distance‑weighted node sampling yields random modules that approximate the internal connectivity of modules, reducing the biases of RandSD (Guney *et al.* 2016).

#### 2.3.3 Statistical significance of inter-module proximity and centrality

A standardized distance was computed by subtracting the mean and dividing by the standard deviation of D(U,W) measured across many randomly generated module pairs of the same sizes as U and W (Fig. 2a–c). Module pairs with significantly smaller distances than expected under this null distribution were defined as crosstalk module pairs, indicating potential biologically meaningful interactions between modules (Fig. 2b).

**Figure 2:**
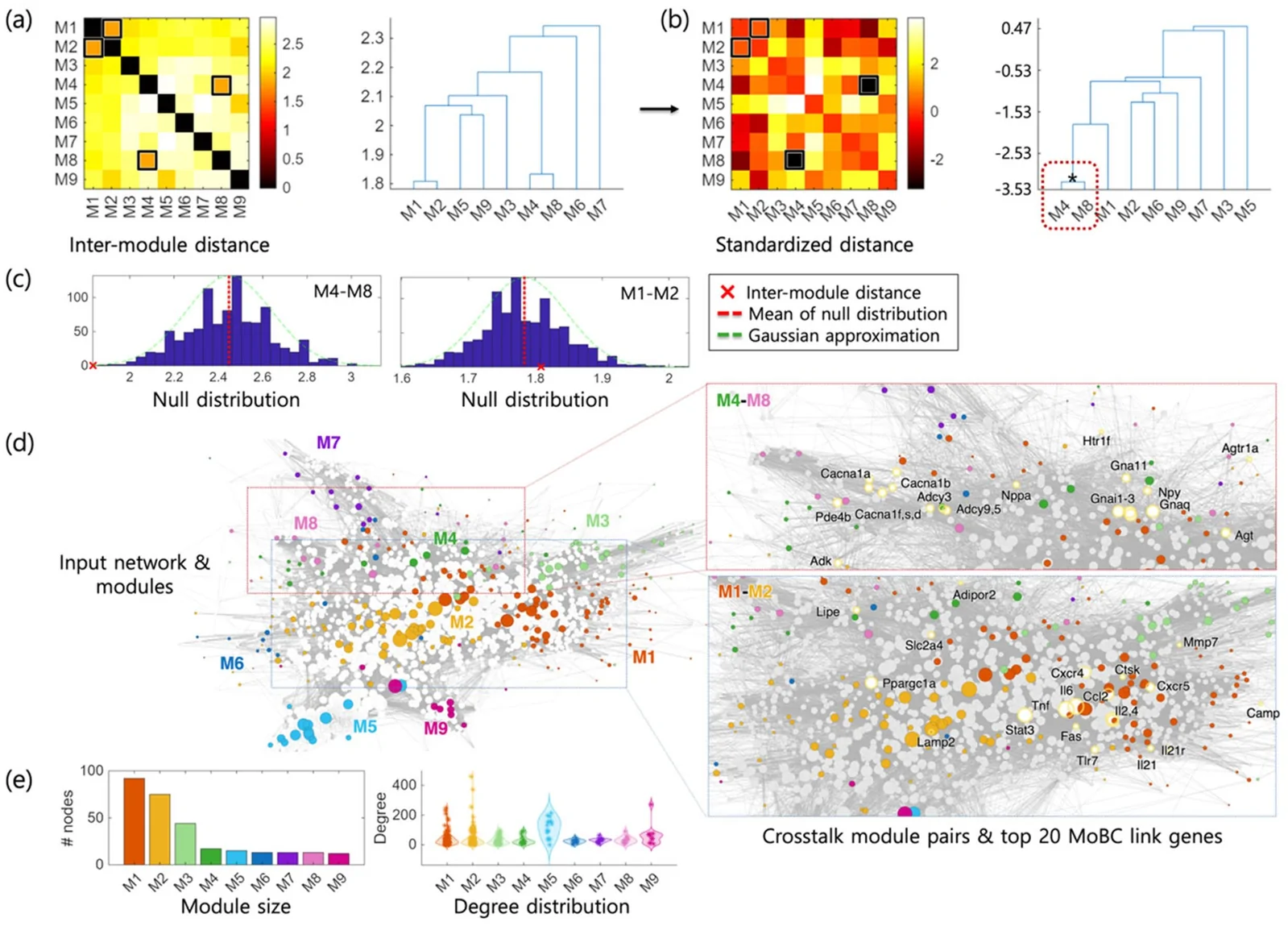
Application of MoBC-Net to the network of CAC data. (a) Closest inter-module distance matrix with the corresponding single-linkage dendrogram (SLD), providing an intuitive overview of relative distances between module pairs. (b) Standardized inter-module distance matrix and SLD. Asterisks (*) indicate statistically significant module pairs ($p_{\mathrm{adjust}}<0.05$). (c) Null distributions of inter-module distances for module pairs $M_{4}$-$M_{8}$ and $M_{1}$-$M_{2}$, generated using the ABin + RandSDM framework. (d) The background network and the nine modules (left). Node colours represent module membership. White nodes outlined in yellow are top-20 prioritized MoBC link genes. The significantly proximal module pair $M_{4}$-$M_{8}$ was selected to identify key link genes mediating their inter-module connectivity. For comparison, key link genes connecting the originally closest module pair, $M_{1}$-$M_{2}$, are also shown. Among the key link genes between $M_{4}$ and $M_{8}$, the more detailed connection structure of Gnai2 is illustrated in Fig. 5d. (e) Module size and degree distributions for the nine modules. $M_{1}$ and $M_{2}$ had larger sizes and higher degrees. Normalization corrected for these biases, and $M_{4}$-$M_{8}$ was identified as the more proximal module pair as shown in (b).

After identifying crosstalk module pairs, we used MoBC to pinpoint key link genes bridging each pair (Fig. 2d). For each crosstalk module pair (U,W), we computed the MoBC score, $c_{\mathrm{MoBC}}^{UW}(v)$, for each candidate link gene v connecting U and W. We then generated random module pairs $\left(U_{\mathrm{rand}},W_{\mathrm{rand}}\right)$ and calculated the corresponding null MoBC scores, $c_{\mathrm{MoBC}}^{U_{\mathrm{rand}}W_{\mathrm{rand}}}(v)$, for the same gene v, in every randomization, assigning zero when v was absent from connecting shortest paths. Empirical p-values for the observed MoBC scores were computed from these null distributions and adjusted for multiple testing using BH false discovery rate (FDR) procedure. Genes with BH-adjusted p-values less than 0.05 that did not belong to either module were considered significant key link genes under the primary criterion. For an expanded prioritization analysis, genes with BH-adjusted P<0.07 were additionally retained, while genes with 0.05≤ BH-adjusted P<0.07 were treated as borderline candidates rather than as meeting the primary 5% FDR criterion. Among these, the top 20 were selected as prioritized MoBC link genes from the expanded set according to $c_{\mathrm{MoBC}}^{UW}(v)$.

Because Monte Carlo significance estimates can be unstable near the significance threshold, RandSDM was initialized with 1000 randomizations and extended in batches of 250 until the prespecified stability criteria were satisfied or a maximum of 5000 randomizations was reached. Empirical p values and BH-adjusted values were recalculated after each batch. The completed adaptive analysis was used to identify significantly close module pairs and significant key link genes. The analysis-specific stopping criteria are described in Supplementary Material Sec. 5, available at supplementary material  *Bioinformatics Advances* online.

### 2.4 Biological relevance assessment

#### 2.4.1 Evaluation of inter-module distance using GO-based semantic similarity

To assess the functional similarity between two modules, we computed GO-based semantic similarities using three complementary measures: Wang, Resnik, and graph information content (GIC) (Gu 2024). GO topology refers to the parent–child connectivity structure of the GO directed acyclic graph. Wang similarity uses semantic contributions propagated through the graph, Resnik similarity uses the information content of the most informative common ancestor. GIC measures information-content sum over common ancestors divided by the sum over the ancestor union. Module-level similarity scores were converted into biological distances as 1 − similarity, so that smaller values indicated greater functional resemblance between modules. Their associations with topological inter-module distances were then evaluated using weighted correlations, with greater weight assigned to module pairs showing stronger RandSDM support for close network proximity. Detailed analyses of semantic similarity and its relationship with topological distance are provided in Supplementary Material Sec. 6, available at supplementary material  *Bioinformatics Advances* online.

#### 2.4.2 Gene set-based evaluation of MoBC link genes

For each module, pathway enrichment analysis was performed using curated gene sets from multiple pathway databases, including KEGG, Reactome, and WikiPathways, as provided in the MSigDB C2 collection (MSigDB v2026.1.Hs) (Liberzon *et al.* 2015). For mouse data, human MSigDB gene sets were used with ortholog mapping to mouse genes. For a given module pair, we selected the top 20 MoBC link genes and examined whether they participated in pathways enriched in both modules (Supplementary Tables 1 and 2, available at supplementary material  *Bioinformatics Advances* online). To evaluate whether this overlap was greater than expected by chance, we generated a null distribution by randomly sampling gene sets from the shortest paths between the modules. Over 1000 iterations, we computed the overlap ratio between each sampled set and the union of enriched pathway gene sets. The empirical distribution of overlap ratios was then used to calculate a P-value for the observed overlap in the top 20 MoBC link genes.

## 3 Results

### 3.1 Module identification and functional annotation

To evaluate the applicability of MoBC-Net to both human and mouse data, we analyzed the transcriptome datasets of TCGA-BRCA and CAC, respectively. Using the 1635 DEGs identified from 224 paired tumour-normal TCGA-BRCA samples, we constructed a DEG-specific PPI network and identified eight modules ($M_{1}$-$M_{8}$) via the Walktrap algorithm. GO enrichment analysis showed that each module was related to distinct biological functions. For example, module $M_{2}$ was enriched for fibroblast growth factor signaling, STAT-mediated receptor signaling and positive regulation of lipid kinase activity, and module $M_{3}$ was associated with extracellular structure organization (Supplementary Material Fig. S14, available at supplementary material  *Bioinformatics Advances* online).

We applied the same approach to the CAC dataset. From 1211 DEGs between cachectic and control mice, we identified nine modules ($M_{1}$-$M_{9}$). GO enrichment revealed coherent functional signatures in each module. For instance, module $M_{4}$ was enriched for positive regulation of cardiac muscle hypertrophy, and module $M_{8}$ was associated with muscle system processes and muscle contraction (Supplementary Material Fig. S15, available at supplementary material  *Bioinformatics Advances* online).

### 3.2 Summary of randomization evaluation and GO-based biological validation

To select an appropriate null model for inter‑module distance and centrality, we systematically compared four combinations of degree‑binning and randomization strategies, TBin + RandSD, TBin + RandSDM, ABin + RandSD and ABin + RandSDM, on the background networks of TCGA-BRCA and CAC (See Sec. 5 in Supplementary Material, available at supplementary material  *Bioinformatics Advances* online). Unlike ABin, TBin uses fixed-size degree bins and does not adapt bin boundaries to module-specific sampling demand (Fig. 4a and d), and yields unsampled bins and hub over‑sampling (Fig. S10c–e, available at supplementary material  *Bioinformatics Advances* online). RandSDM consistently generated random modules with higher modularity (Fig. 4b and e) and smaller diameters than RandSD (Fig. 4c and f, available at supplementary material  *Bioinformatics Advances* online). Based on these experiments, we adopted ABin with RandSDM as our default randomization scheme. We set κ=0.07 for ABin because it produced sampling frequencies and degree distributions comparable to TBin with τ=100, as reported in previous studies (Supplementary Fig. S10 in Sec. 5, available at supplementary material  *Bioinformatics Advances* online) (Guney *et al.* 2016). A more detailed parameter-sensitivity and diagnostic evaluation of the binning and randomization schemes is provided in Supplementary Sec. 5, available at supplementary material  *Bioinformatics Advances* online.

We evaluated whether randomization-based normalization reduced module size- and hubness-related biases in inter-module distance. Hubness was defined as the mean degree of the top 5% highest-degree nodes in each module pair. Raw inter-module distance was negatively correlated with hubness in both TCGA-BRCA and CAC, and with module size especially in CAC. It indicates that large or hub-enriched module pairs can appear artificially close (Table 1). After normalization, these negative correlations were attenuated in CAC across all four binning and randomization schemes.

**Table 1:** Correlations of raw and standardized MoBC-based inter-module distances with module hubness and module-pair size across randomization schemes.

Correlation		TCGA-BRCA		CAC	
		Deg.	Size	Deg.	Size
**Inter-module distance**		−0.42	−0.14	−0.46	−0.67
**Standardized**	**RandSD + TBin**	0.13	0.52	−0.11	−0.33
	**RandSDM + TBin**	0.11	0.52	−0.10	−0.33
	**RandSD + ABin**	0.13	0.52	−0.11	−0.33
	**RandSDM + ABin**	0.11	0.52	−0.09	−0.31

We then compared MoBC with S2B and FCS under the selected RandSDM-based framework (Table 2). The null distribution mean and standard deviation were strongly associated with module size and hubness, supporting the need for pair-specific normalization. In CAC, MoBC normalization reduced size- and hubness-related correlations more effectively than S2B or FCS, which retained stronger negative correlations after normalization. In TCGA-BRCA, the positive size correlation after MoBC normalization suggests that larger module pairs were not preferentially assigned closer-than-random scores. It indicates a conservative effect for larger modules. Finally, we examined biological concordance using weighted correlations with GO-based semantic distances (Table 3 and Supplementary Material Fig. S13 and Table S6, available at supplementary material  *Bioinformatics Advances* online). MoBC showed the strongest association with semantic distances in TCGA-BRCA, whereas S2B and FCS showed stronger associations in CAC.

**Table 2:** Correlations of raw distance, standardized distance, and RandSDM null-distribution statistics with module hubness and module-pair size for MoBC, S2B, and FCS.

Method			TCGA-BRCA		CAC	
			Deg.	Size	Deg.	Size
**MoBC**	**Distance**		**−0.42**	−0.14	**−0.46**	**−0.67**
	**Standardized**		0.11	0.52	−0.09	−0.31
	**Null**	**Mean**	**−0.82**	**−0.62**	**−0.76**	**−0.48**
		**Std.**	**−0.82**	**−0.96**	**−0.47**	**−0.94**
**S2B**	**Distance**		−0.28	−0.08	**−0.55**	**−0.50**
	**Standardized**		0.09	0.25	**−0.45**	**−0.66**
	**Null**	**Mean**	**−0.60**	**−0.38**	**−0.60**	0.14
		**Std.**	**−0.43**	**−0.65**	**−0.47**	**−0.93**
**FCS**	**Distance**		−0.22	0.03	**−0.58**	**−0.47**
	**Standardized**		−0.16	0.16	**−0.48**	**−0.65**
	**Null**	**Mean**	**−0.43**	−0.25	**−0.56**	0.22
		**Std.**	**−0.89**	**−0.91**	**−0.57**	**−0.94**

Boldface indicates nominal significance at *P* < 0.05.

**Table 3:** Weighted correlations between topological inter-module distances and GO-based semantic distances.

TCGA-BRCA	Wang	Resnik	GIC
**MoBC**	**0.73**	**0.78**	**0.81**
**S2B**	0.01	−0.06	−0.05
**FCS**	0.17	−0.01	0.00

CAC	Wang	Resnik	GIC
**MoBC**	0.31	0.41	0.21
**S2B**	**0.68**	**0.60**	**0.57**
**FCS**	**0.68**	**0.60**	**0.54**

Correlations were calculated using mean standardized distances and mean raw RandSDM *P*-value weights across three independent randomization runs. Boldface indicates nominal significance at *P *< 0.05.

Together, these analyses support ABin + RandSDM as a topology-aware null model. It preserves degree-related constraints while partially retaining local modular structure, allowing pair-specific normalization of inter-module proximity. Under this framework, MoBC behaved as a conservative crosstalk measure. It reduced size- and hubness-related bias more effectively than S2B/FCS in CAC and showed the strongest semantic concordance in TCGA-BRCA. These results suggest that MoBC can prioritize crosstalk module pairs based on statistical robustness or biological interpretability, depending on the dataset.

### 3.3 Selection of crosstalk module pair

In the network of TCGA-BRCA, the pair of modules $M_{2}-M_{3}$ was the closest under both raw and standardized distance metrics (BH-adjusted P<0.05), as well as under the GO-based distance. Accordingly, we chose this pair as the crosstalk module pair for the MoBC analysis. In the network of CAC, raw and GO-based distance metrics ranked $M_{1}$ and $M_{2}$ as the closest pair. However, since $M_{1}$ and $M_{2}$ were the largest and highest-degree modules among the nine CAC modules (Fig. 2e), the closest pair was changed to $M_{4}-M_{8}$ after normalization (BH-adjusted P<0.05, Fig. 2a and b). $M_{1}$ and $M_{2}$ were associated with JAK-STAT signaling pathway as well as cytokine signaling and FoxO signaling pathways of which roles were critical in CAC (Sandri *et al.* 2004, Bonetto *et al.* 2012). Therefore, we examined not only the pre‑normalization closest pair $M_{1}-M_{2}$ but also the standardized closest pair $M_{4}-M_{8}$ in the network of CAC to identify its key link genes (Fig. 2d).

We also applied MoBC-Net to a weighted gene coexpression network analysis (WGCNA)-based background network as an additional sensitivity analysis (Supplementary Material Sec. 7B, available at supplementary material  *Bioinformatics Advances* online). However, the denser co-expression network produced larger and more consolidated modules, and no robust MoBC-significant crosstalk pairs were detected after RandSDM normalization. These results support that MoBC yielded more selective and biologically interpretable crosstalk module pairs in the PPI-based disease subnetworks than in the denser co-expression network (Supplementary Material Sec. 2C, available at supplementary material  *Bioinformatics Advances* online).

### 3.4 Inferring key link genes between crosstalk module pairs using MoBC

We applied MoBC-Net to all pairs of modules to investigate link genes that were significantly over-represented across the shortest paths between the closest nodes within each pair. To assess the biological relevance of the link genes identified in each pair, we assessed whether these link genes participated in the biological pathways associated with the corresponding modules. We assumed that if two modules are enriched in the same biological pathways, then genes connecting them are likely to be involved in those pathways as well. Based on this assumption, functional relevance was evaluated by quantifying the involvement of link genes in biological pathways shared between the two modules and by determining whether this overlap was significantly greater than that observed in randomly sampled gene sets (see Methods and Fig. 3a).

**Figure 3:**
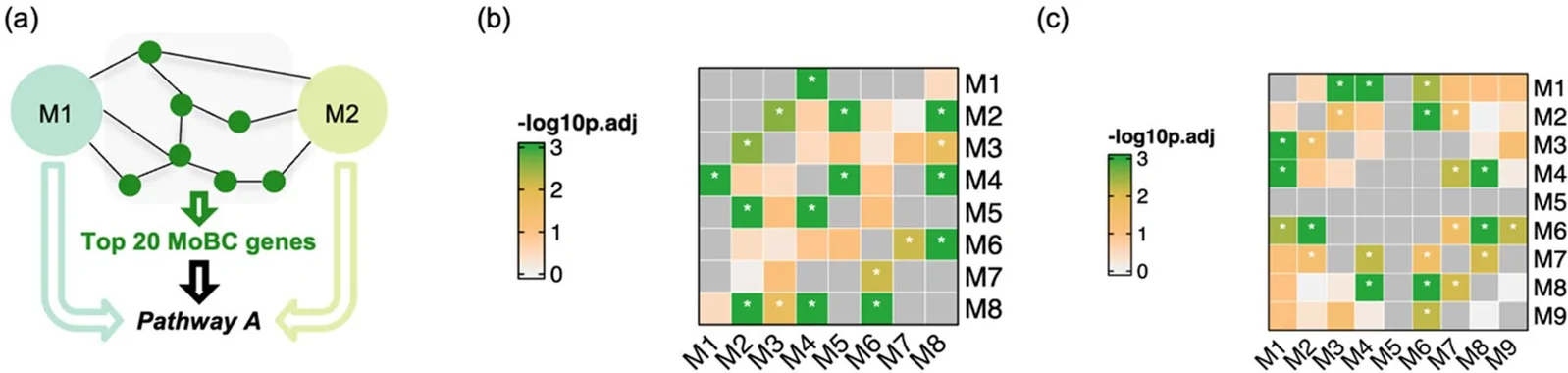
Association analysis of MoBC link genes with their respective modules based on curated gene sets from pathway databases. (a) The graphical abstract provides an overview of the analysis. (b, c) The heatmap shows the enrichment significance of the top 20 link genes in shared pathways between modules in the TCGA-BRCA (b) and CAC (c) datasets. The white star indicates significant enrichment of the MoBC link genes relative to random genes ($p_{\mathrm{adjust}}<0.05$). The colors of heatmap indicate enrichment significance.

**Figure 4:**
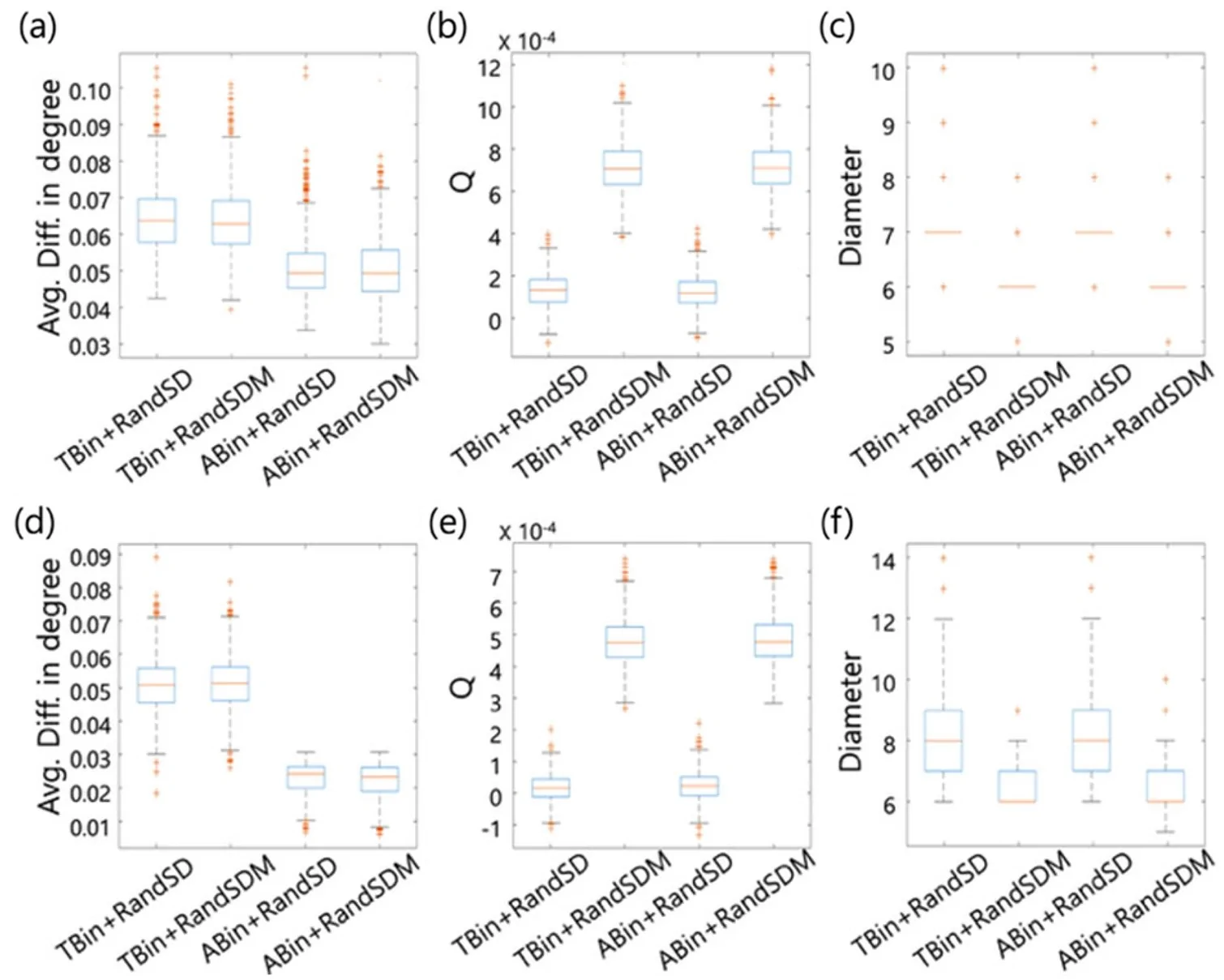
Comparison of randomization methods for TCGA-BRCA (a–c) and CAC (d–f). (a, d) Average differences in degree distribution between the original and randomized disease gene modules. (b, e) Modularity Q. (c, f) Diameter of randomized gene modules.

In the TCGA‑BRCA data, 19 module pairs shared at least one pathway. In nine of these, the MoBC link genes showed significantly greater overlap with the shared pathway genes than randomly selected genes, including closely positioned module pairs such as $M_{2}-M_{3}$ (BH-adjusted P<0.05) (Fig. 3b). In cancer cachexia, 24 module pairs shared at least one significantly enriched pathway, and 12 of these pairs showed significant overlap between the link genes and the shared gene set, including the adjacent module pair $M_{4}-M_{8}$, whereas $M_{1}-M_{2}$ did not (Fig. 3c). These findings support the hypothesis that MoBC link genes act as functional bridges between modules. We then examined representative cases in depth. In TCGA-BRCA, modules $M_{2}$ and $M_{3}$ were linked via the PI3K–Akt pathway, with seven MoBC link genes (TLR4, COL2A1, TLR2, FGF23, EPHA2, VTN, and AREG) mapping to this pathway (Fig. 5a). Among these link genes, TLR4 has been reported to play a crucial role in the growth, invasion and metastasis of breast cancer cells (Kathirasan *et al.* 2024). In addition, consistent with the known roles of EPHA2 in EGFR-mediated tumorigenesis and extracellular matrix regulation, our network analysis showed that EPHA2 connected EGFR in $M_{2}$ with MMP10 in $M_{3}$ (Fig. 5b) (Zelinski *et al.* 2001, Xiao *et al.* 2020, Vaught *et al.* 2021, Zhao *et al.* 2021, Kim *et al.* 2022).

**Figure 5:**
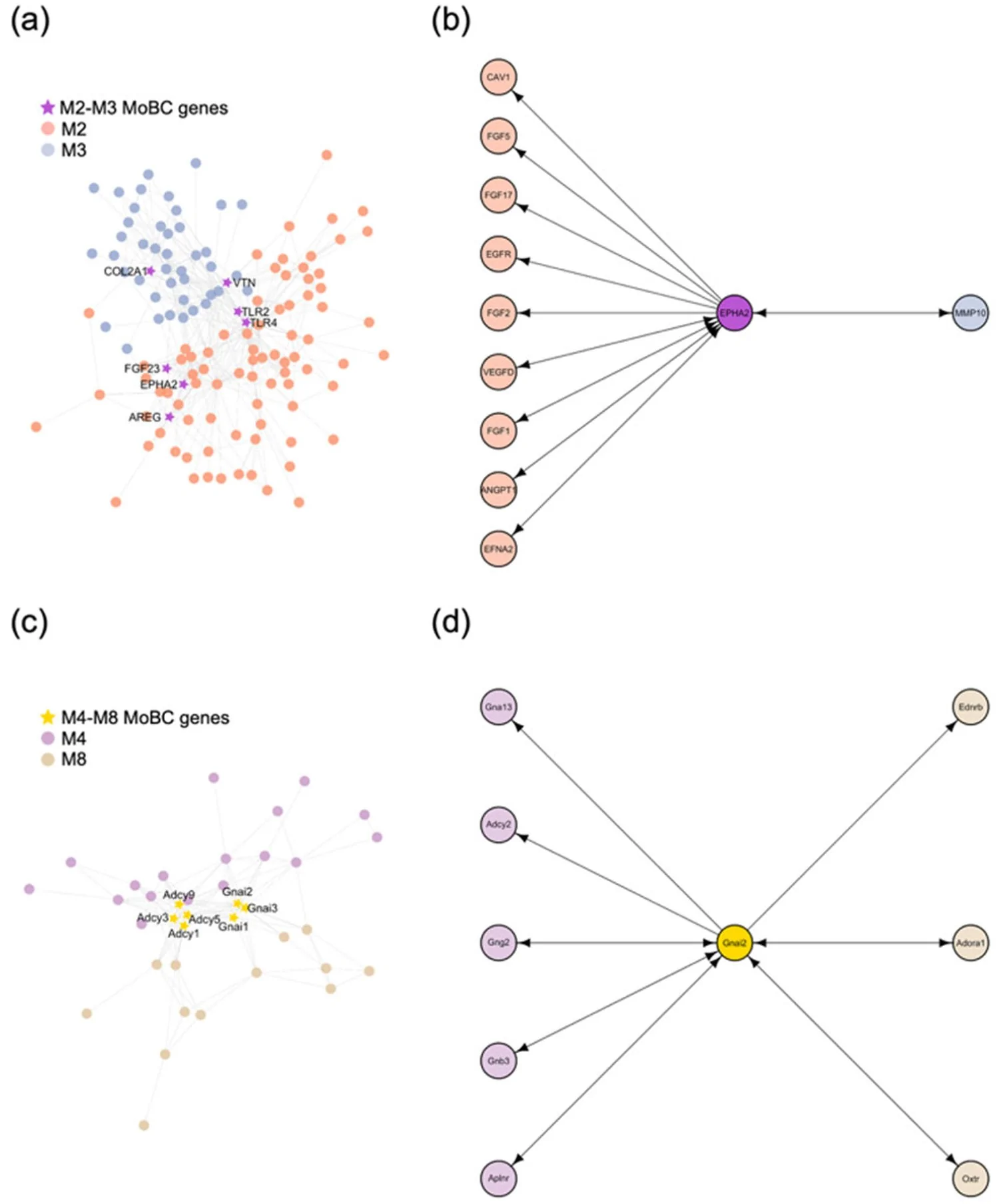
Crosstalk module pair in the background network and the paths of a key link gene from TCGA-BRCA and CAC datasets. (a) The subnetwork shows interactions between TCGA-BRCA modules $M_{2}$ (pink) and $M_{3}$ (blue). Top MoBC link genes involved in the PI3K-Akt signaling pathway are marked with purple stars. (b) The directed-path graph highlights EPHA2 (purple) as the key link gene; arrows show the directed shortest path between $M_{2}$ (pink) and $M_{3}$ (blue) nodes via EPHA2. (c) The subnetwork shows interactions between CAC modules $M_{4}$ (light purple) and $M_{8}$ (beige). Top MoBC link genes involved in the apelin and cAMP signaling pathway are marked with yellow stars. (d) The directed-path graph highlights Gnai2 (yellow) as the key MoBC link gene; arrows show the directed path between $M_{4}$ (light purple) and $M_{8}$ (beige) nodes via Gnai2.

To further assess protein-level relevance of the link genes, we analyzed an independent CCLE/DepMap BRCA cell-line proteome dataset (Supplementary Material Sec. 9 and Fig. S23, available at supplementary material  *Bioinformatics Advances* online) (Nusinow *et al.* 2020). Several link genes showed subtype-associated protein-abundance patterns, especially in basal A and/or basal B subtypes. CD44 showed high protein abundance in basal A/B subtypes, whereas EPHA2 protein abundance was lower in luminal and HER2-amplified subtypes but relatively maintained in basal-like cell lines.

In the network of CAC, MoBC-Net identified members of the Gnai family (Gnai1, Gnai2, and Gnai3) and the Adcy family (Adcy1, Adcy3, Adcy5, and Adcy9) as link genes between modules $M_{4}$ and $M_{8}$, all of which were involved in both the cAMP and apelin signaling pathways (Fig. 5c). Notably, among these link genes, Gnai2, which has been reported to promote muscle hypertrophy (Minetti *et al.* 2011), was identified by MoBC-Net as a connector linking the apelin receptor (Aplnr) in $M_{4}$ to Adora1 in $M_{8}$ (Fig. 5d). Additionally, between modules $M_{1}$ and $M_{2}$, MoBC-Net identified Il6 as the only link gene within the shared JAK–STAT pathway (Supplementary Material Fig. S21(a), available at supplementary material  *Bioinformatics Advances* online). Il6 has been reported as a key mediator of cachexia‑induced muscle wasting by activating FoxO signaling pathway (Setiawan *et al.* 2023, Wang *et al.* 2023). In particular, MoBC-Net showed that Il6 connected cytokines in $M_{1}$ to FoxO and JAK-STAT signaling pathway genes in $M_{2}$ (Supplementary Material Fig. S21(b), available at supplementary material  *Bioinformatics Advances* online) (Setiawan *et al.* 2023, Wang *et al.* 2023).

Further details on the key-link gene analysis and prioritized genes are provided in Supplementary Material Sec. 7D, available at supplementary material  *Bioinformatics Advances* online. We also compared MoBC-based key link gene prioritization with S2B and FCS in the Supplementary Material Sec. 8B, available at supplementary material  *Bioinformatics Advances* online. The key link genes identified by MoBC showed broadly consistent rankings with those obtained by S2B and FCS (Supplementary Material Fig. S22, available at supplementary material  *Bioinformatics Advances* online). However, MoBC required markedly less runtime per randomization iteration than S2B or FCS, especially for larger module pairs (Supplementary Material Table S5, available at supplementary material  *Bioinformatics Advances* online). Thus, MoBC provides a practical compromise for downstream key link gene analysis by preserving concordant prioritization with existing path-based measures while remaining more scalable for repeated null-model testing.

## 4 Discussion and conclusions

MoBC-Net was developed to support mechanism-oriented interpretation of disease networks in which DEGs form multiple functionally coherent subnetworks rather than a single homogeneous disease module. In this setting, the relevant question is not only which genes are differentially expressed or highly connected, but also which genes connect disease-associated modules in the broader interaction network. MoBC-Net addresses this distinction by first defining DEG-associated modules and then testing whether module pairs are closer than expected under a null model that matches module size and degree distribution while favoring local compactness. Candidate link genes are subsequently prioritized based on shortest-path interfaces between statistically supported module pairs. Thus, MoBC-Net is intended to identify module-level connectors, including genes not identified as DEGs whose relevance may not be evident from transcript-level differential expression alone.

The use of shortest paths is central to this framework. Shortest paths should not be interpreted as literal biochemical signaling routes, but as compact topological representations of potential communication between molecular neighborhoods. This rationale is supported by recent studies showing that shortest-path-preserving metric or distance backbones can retain shortest-path distributions and community structure, and by PPI-based biological analyses in which backbone-like shortest-path structures uncovered conserved regulatory programs and candidate disease genes (Brattig Correia *et al.* 2023, Brattig-Correia *et al.* 2024, Dreveton *et al.* 2024). MoBC-Net does not extract a global metric backbone; instead, it applies a related principle at the module-pair level by evaluating shortest paths crossing disease-module boundaries against pair-specific randomized modules.

This interpretation should be distinguished from correcting all biases in PPI databases. Existing PPI networks can over-represent well-studied proteins and interactions, and MoBC-Net does not remove these database-level biases (Gillis *et al.* 2014, Cafarelli *et al.* 2017). The specific bias addressed here is narrower and topological: large or hub-enriched modules can appear close simply because they contain many possible endpoints. The ABin + RandSDM procedure controls for this effect by comparing each observed module pair with randomized module pairs matched for size and degree distribution, while approximating local compactness. Therefore, a significant standardized MoBC distance should be interpreted as evidence that two modules are closer than expected for modules with comparable topology, not as proof that the identified link genes are causal regulators. Among the evaluated null models, ABin combined with RandSDM provided the most informative compromise for the present task. ABin improved degree-class sampling coverage, whereas RandSDM generated random modules whose cohesion and diameter more closely approximated those of the observed modules than RandSD (Supplementary Material Sec. 5 and Figs. S10–S11, available at supplementary material  *Bioinformatics Advances* online). These results suggest that an informative null model should match module size and degree composition, approximate local cohesion, and disrupt the specific proximity and connector structure of the observed module pair. ABin + RandSDM does not model edge weights, higher-order topology, or condition-specific interactions. Incorporating such features would be an important direction for future null-model development.

The TCGA-BRCA analysis illustrates how MoBC-Net can refine disease-mechanism hypotheses at the module-interface level. The top 20 $M_{2}-M_{3}$ link genes suggested a functional connection between PI3K–Akt signaling and extracellular matrix (ECM)-associated tumor biology (Table S1, available at supplementary material  *Bioinformatics Advances* online). An independent analysis of breast cancer cell lines from the CCLE/DepMap datasets revealed subtype-associated protein-abundance patterns that provided complementary context for interpreting these transcriptome-derived candidates (Sec. 3.4; Supplementary Material Sec. 9 and Fig. S23, available at supplementary material  *Bioinformatics Advances* online). Among these candidates, EPHA2 was connected to CD44, TGFB1, SHC1, LOX, MMP2, and VTN, suggesting a receptor tyrosine kinase (RTK)–epithelial-to-mesenchymal transition (EMT)–ECM remodeling axis consistent with the mesenchymal and invasive characteristics of basal-like breast cancer models (Dai *et al.* 2017, Fattet *et al.* 2020). Together, these findings indicate that $M_{2}-M_{3}$ link genes capture a broader subtype-associated program involving PI3K–Akt signaling, EMT-related invasion, and ECM remodeling, rather than an isolated single-gene signal.

The cancer cachexia analysis provides a complementary example in which normalization changed the module pair prioritized for downstream interpretation. Before normalization, the $M_{1}-M_{2}$ pair was closest, consistent with the established involvement of cytokine, JAK–STAT, and FoxO-related mechanisms in cachexia. After RandSDM normalization, however, $M_{4}-M_{8}$ emerged as the closest module pair. This pair connected a cAMP/GPCR-related module with a calcium/contractile signaling module, and its link genes included Gnai and Adcy family members. Gnai2 is biologically relevant because Gαi2 signaling has been reported to promote skeletal muscle hypertrophy, myoblast differentiation, and muscle regeneration (Minetti *et al.* 2011). Recent evidence also implicates impaired cAMP–PKA–CREB1 signaling in mitochondrial dysfunction and skeletal muscle wasting during cancer cachexia (Angelino *et al.* 2025). Thus, the standardized $M_{4}-M_{8}$ result should not be interpreted as replacing the canonical IL-6/JAK–STAT/FoxO axis represented by $M_{1}-M_{2}$. Rather, it suggests an additional cAMP/GPCR–contractile signaling interface that becomes visible after correcting for module size and hubness.

An important limitation is that MoBC-Net depends on both the background network and the module-detection method. Module detection is not a neutral preprocessing step, because MoBC-Net can only test crosstalk across module boundaries that remain separated in the input partition. Leiden and Louvain produced strong modularity-oriented and enrichment-consistent partitions, but their broader modules merged the Walktrap $M_{2}-M_{3}$ pair in TCGA-BRCA and the Walktrap $M_{4}-M_{8}$ pair in CAC into single larger modules (Supplementary Material Sec. 7C and Table S8, available at supplementary material  *Bioinformatics Advances* online). Once such boundaries are collapsed, the corresponding non-DEG link genes can no longer be prioritized as inter-module connectors, even though they may provide mechanistic insight into disease-module interactions. This does not imply that Leiden or Louvain are inferior community-detection methods, but different algorithms expose different biological resolutions. Broader modularity-oriented partitions may be useful for summarizing large functional programs, whereas MoBC-Net requires sufficiently fine-grained and locally cohesive modules to identify local disease-module interfaces. This is consistent with the known resolution dependence of modularity-based community detection (Fortunato and Barthélemy 2007, Traag *et al.* 2019). In the PPI-based analyses performed here, Walktrap was therefore appropriate as the primary module-detection method because its short random-walk scale preserved compact local module boundaries suitable for downstream MoBC-Net analysis. Although this comparison covered representative flat community-detection algorithms, stochastic block models and multiresolution consensus/hierarchical clustering were not included as primary comparators because their outputs may require additional interpretation or hierarchy-cutting before they can be used as MoBC-Net input modules. Future applications could incorporate such partitions by first selecting biologically interpretable, non-overlapping, sufficiently sized, and locally cohesive flat modules, followed by the same cohesion, enrichment, stability, and sensitivity checks used here for Walktrap, Leiden, Louvain, and Infomap (Supplementary Material Sec. 3, available at supplementary material  *Bioinformatics Advances* online).

MoBC-Net was also sensitive to the choice of background network. The PPI networks analyzed here were sparse interaction backbones in which local bridge structures remained visible after RandSDM standardization. In contrast, the corrected BRCA-WGCN was denser and more consolidated. No selected BRCA-WGCN partition contained a significantly close MoBC pair, suggesting that dense and redundant connectivity may reduce the detectability of local bridge structures. Network sparsification or shortest-path-preserving backbone extraction may therefore be useful for dense co-expression networks (Brattig Correia *et al.* 2023). However, sparsification was not evaluated in the present study, and it remains to be determined whether it would recover interpretable MoBC relationships without distorting the co-expression structure. Future applications should therefore examine sparsification or backbone extraction together with network density, module granularity, parameter sensitivity, and independent biological validation.

Taken together, MoBC-Net provides a multi-scale, module-aware framework that prioritizes inter-module crosstalk and candidate link genes, complementing conventional DEG and pathway enrichment analyses.

## Supplementary Material

vbag279_Supplementary_Data

## Data Availability

An open-source R implementation of the MoBC-Net, together with the data used in this study, is available at GitHub at: https://github.com/mogam-ai/MoBC-Net.
