# Supplementary material for: MoBC-Net: computing network inter-module distance and module-based betweenness centrality
Source: Bioinform Adv. 2026 Oct 3;6(1):vbag279. doi: 10.1093/bioadv/vbag279 (PMC13722279; doi:10.1093/bioadv/vbag279)
Supplement: Supplementary file 1 [file vbag279_supplementary_data.zip › draft.mobcnet.supp.20260819.pdf]

Supplementary material for "MoBC-Net: Computing Network Inter-module Distance and Module-based Betweenness Centrality"

1. DEFINITIONS, NOTATION, AND ASSUMPTIONS

Let $G = (V, E)$ be an undirected background network and let d_{uw} be its unweighted shortest-path distance between nodes u and w. The *background network* is the complete PPI or co-expression network used to calculate shortest paths and generate random modules. A *DEG-overlap subnetwork* is the graph induced by DEG nodes mapped to G and by the edges connecting those nodes. Community detection is performed on the DEG-overlap subnetwork, and inter-module distance, randomization, and candidate link-gene scoring are evaluated in the background network.

For the parameter setting of a community detection method (for example, Leiden at resolution 0.7), $\mathcal{M} = \{M_1, \ldots, M_K\}$ denotes the resulting non-overlapping modules. Modules are numbered after sorting by decreasing size. A module contains more than 20 nodes for BRCA-PPI and BRCA-WGCN or more than 10 nodes for CAC-PPI. A setting is eligible for parameter selection when $K \geq 5$, giving at least ten module pairs for downstream correlation and module-label permutation analyses.

MoBC-Net accepts any non-overlapping module partition whose nodes occur in the background network. The community-detection method and parameter determine the granularity of the partition. Gene groups assigned to the same module are treated as within-module structure, whereas groups assigned to different modules remain available for subsequent inter-module distance analysis. A candidate key link gene has positive observed MoBC, occurs outside both focal modules, and lies on an eligible shortest path connecting the two modules. Unless otherwise stated, a *significantly closest pair* is the minimum observed-distance pair among those with one-sided lower-tail RandSDM Benjamini–Hochberg (BH)-adjusted $p < 0.05$ in the stated correction scope.

2. BACKGROUND NETWORKS AND DEG-OVERLAP SUBNETWORKS

A. Differential expression analysis

To prioritize the robust DEGs of BRCA, we applied stringent cutoffs of BH-adjusted $p < 0.01$ and $|\log_2 \mathrm{FC}| > 2$, since a large proportion of genes exhibited relatively small fold changes. This conservative filtering strategy was used to retain only genes with strong differential expression signals. This yielded 1,635 BRCA DEGs: 878 upregulated and 757 downregulated.

The DEGs of CAC were defined as genes with BH-adjusted $p < 0.05$ and $|\log_2 \mathrm{FC}| > 1$. A stricter fold-change threshold would have substantially reduced the number of identified DEGs. Therefore, the conventional $|\log_2 \mathrm{FC}| > 1$ cutoff was used to retain sufficient biologically relevant genes. This yielded 1,211 CAC DEGs: 680 upregulated and 531 downregulated.

The Minus-Average (MA) and volcano plots are shown in Figure S1.

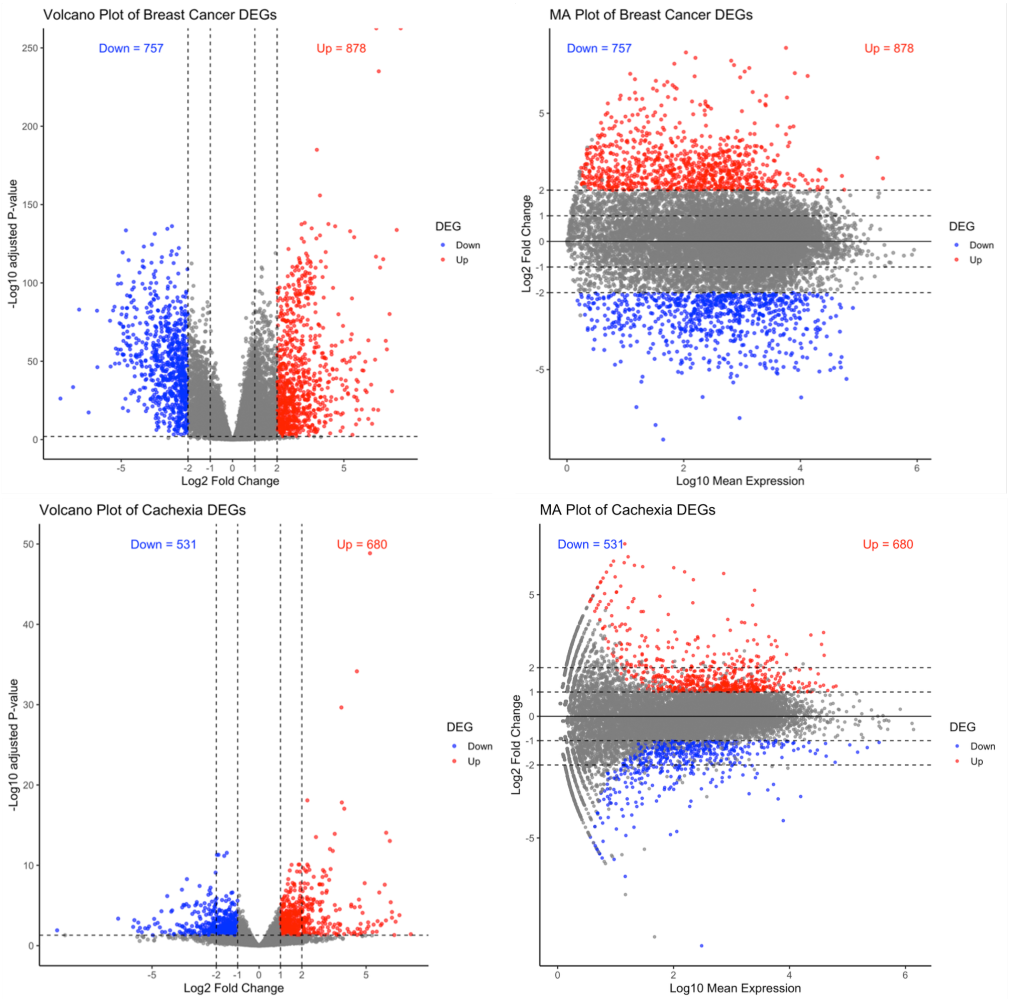

Fig. S1. Differential expression filters used to define DEG-overlap subnetworks. Top: BRCA, bottom: CAC. Dashed vertical or horizontal lines indicate the network-specific fold-change and adjusted-p thresholds.

B. Network specifications

Table S1 summarizes the giant background components and the DEG-overlap graphs used in the analyses. BRCA-WGCN was constructed by thresholding the absolute BRCA expression-correlation matrix at 0.80. Its background giant component contained 4,965 nodes and 80,404 edges. Of the 1,635 BRCA DEGs, 795 mapped to this component. After construction of the analyzable DEG-overlap giant component, 794 nodes and 31,794 edges remained.

Table S1. Background networks and DEG-overlap subnetworks.

Network	Background nodes	Background edges	DEGs	DEG-subnetwork nodes	DEG-subnetwork edges	Module-size filter
BRCA-PPI	15,867	235,783	1,635	1,344	5,775	$\lvert M\rvert > 20$
CAC-PPI	12,543	169,320	1,211	902	1,162	$\lvert M\rvert > 10$
BRCA-WGCN (0.80)	4,965	80,404	1,635	794	31,794	$\lvert M\rvert > 20$

The absolute-correlation threshold was evaluated over 0.70–0.95. A threshold of 0.80 balanced connected coverage, community structure, small-worldness, path length, core density, and scale-free fit while avoiding both the very dense low-threshold graphs and the fragmented high-threshold graphs (Figure S2).

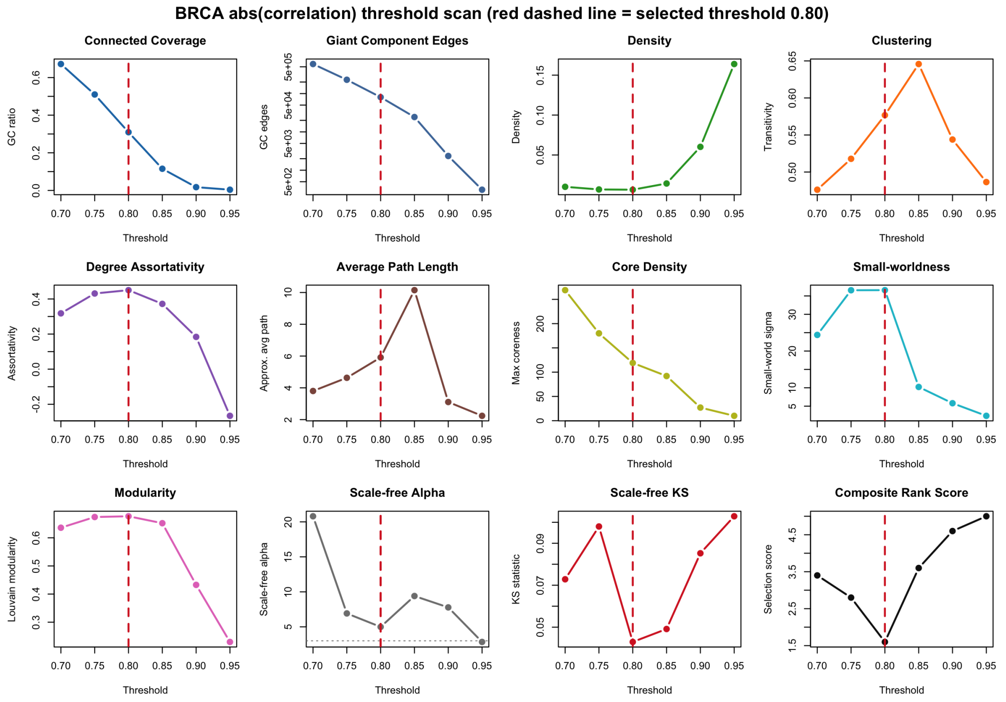

Fig. S2. BRCA absolute-correlation threshold scan. The red dashed line denotes the selected threshold of 0.80.

C. Comparison of background networks

The three background networks showed different descriptive topological patterns (Table S2). Compared with the two PPI networks analyzed here, the BRCA-WGCN had higher edge density, transitivity, and degree assortativity. Under the Leiden partition used for this descriptive comparison, the BRCA-WGCN also had fewer communities, a larger largest-community share, a higher Gini coefficient, and a lower effective number of communities. Together with its higher mean within-community density and lower mean conductance, these measures were consistent with a more locally clustered, internally cohesive, and unevenly distributed community organization. In contrast, the BRCA-PPI and CAC-PPI networks had lower edge density and more evenly distributed community sizes. The BRCA-WGCN nevertheless had a longer average shortest-path length, showing that its greater local connectivity was not accompanied by uniformly shorter paths across the network.

Table S2. Topological comparison of the three background networks. Metrics were calculated on the simplified giant component of each network. Leiden modularity and community-level summaries were obtained using `igraph` with the modularity objective.

Network	Density	Trans.	Assort.	Avg. path	Max core	Leiden Q	#comm.	Largest share	Gini	Eff. comm.	Mean density	Mean cond.	Coverage	Performance
BRCA-PPI	0.0019	0.342	0.281	3.94	112	0.690	31	0.127	0.619	15.37	0.198	0.182	0.787	0.924
CAC-PPI	0.0022	0.388	0.368	4.13	107	0.724	29	0.117	0.615	14.43	0.234	0.164	0.819	0.920
BRCA-WGCN	0.0065	0.577	0.450	5.86	119	0.677	25	0.274	0.715	9.53	0.255	0.100	0.859	0.856

3. COMMUNITY-DETECTION PARAMETER SENSITIVITY AND SELECTION

A. Methods and parameter grids

We evaluated Walktrap, Leiden, Louvain, and Infomap [1, 9, 11, 12]. Walktrap was implemented using `igraph::cluster_walktrap()` with 3–9 random-walk steps. Leiden was implemented using `igraph::cluster_leiden()` with the modularity objective, 10 iterations, and resolution values of 0.6, 0.7, 0.8, 0.9, 1.0, 1.1, 1.2, 1.5, 2.0, 3.0, and 4.0. Louvain was implemented using `igraph::cluster_louvain()` with the same resolution grid. Infomap was implemented using `bioregion::netclu_infomap()` for an unweighted, undirected network with two-level cluster-

ing and Markov times of 0.25, 0.5, 0.75, 1.0, 1.5, 2.0, and 4.0. This produced 36 method–parameter settings per network and 108 settings overall (Table S3).

Table S3. Expanded community-detection parameter grid.

Method	Parameter	Values	Interpretation
Walktrap	steps	3, 4, 5, 6, 7, 8, 9	Length of the local random walk used during agglomeration
Leiden	resolution	0.6–1.2 by 0.1; 1.5, 2, 3, 4	Granularity of modularity optimisation
Louvain	resolution	0.6–1.2 by 0.1; 1.5, 2, 3, 4	Granularity of modularity optimisation
Infomap	Markov time	0.25, 0.5, 0.75, 1, 1.5, 2, 4	Effective flow-resolution scale

B. Six component scores

Each method–parameter setting was evaluated using six complementary component scores: size appropriateness, graph quality, background-network cohesion, enrichment consistency, stability, and reproducibility. All scores were scaled from 0 to 100, with higher values indicating a more favorable partition. These components assess different properties of module detection: module-size interpretability, structural organization in the DEG-overlap graph, preservation of module cohesion in the full background network, functional interpretability, robustness to the tuning parameter, and robustness to input-data and random-seed perturbations, respectively.

Let

$$G_{\mathrm{DEG}} = (V_{\mathrm{DEG}}, E_{\mathrm{DEG}})$$

denote the DEG-overlap network and

$$G_{\mathrm{BG}} = (V_{\mathrm{BG}}, E_{\mathrm{BG}})$$

the corresponding full background network. For a method-specific parameter value θ, let

$$\mathcal{M}^{(\theta)} = \left\{ M_1^{(\theta)}, \ldots, M_{K_\theta}^{(\theta)} \right\}$$

denote the modules passing the downstream minimum-size filter. Modules containing more than 20 genes were analyzed in BRCA-PPI and BRCA-WGCN, whereas modules containing more than 10 genes were analyzed in CAC-PPI.

Size appropriateness. Size appropriateness quantifies whether the analyzed modules have balanced and interpretable sizes without being dominated by one disproportionately large module. Let

$$s_i = |M_i|, \qquad L = \frac{\max_i s_i}{\sum_{i=1}^{K} s_i},$$

where K is the number of analyzed modules and L is the fraction of module-assigned genes contained in the largest module.

For module sizes sorted in ascending order, $s_{(1)} \leq \cdots \leq s_{(K)}$, the Gini coefficient was calculated as

$$Gini = \frac{2\sum_{i=1}^{K} i s_{(i)}}{K\sum_{i=1}^{K} s_{(i)}} - \frac{K+1}{K}.$$

Because a smaller Gini coefficient represents a more balanced size distribution, the score uses the balance-transformed term $1 - Gini$.

The interquartile-range balance term was defined as

$$B_{\mathrm{IQR}} = 1 - \mathrm{clip}_{[0,1]}\left\{ \frac{\mathrm{IQR}(s_1, \ldots, s_K)}{\max_i s_i} \right\},$$

where $\mathrm{clip}_{[0,1]}(x) = \max(0, \min(1, x))$. This term approaches one when the central portion of the module-size distribution is narrow relative to the largest module.

Dominance by the largest module was penalized using

$$f_{dom}(L) = \begin{cases} 1, & L \leq 0.35, \\ 1 - \dfrac{L - 0.35}{0.40}, & 0.35 < L < 0.75, \\ 0, & L \geq 0.75. \end{cases}$$

Thus, no penalty is applied when the largest module contains at most 35% of module-assigned genes, whereas the contribution becomes zero when it contains 75% or more.

An explicit module-count prior was included to favor the level of granularity considered interpretable for the downstream analysis:

$$C(K) = \exp\left[-\frac{(K-K_0)^2}{2(1.5)^2}\right].$$

Here, $K_0 = 8$ for BRCA-PPI and BRCA-WGCN and $K_0 = 9$ for CAC-PPI.

The complete size-appropriateness score was

$$S_{\text{size}} = 100\left\{0.20(1 - Gini) + 0.20B_{\text{IQR}} + 0.30f_{dom}(L) + 0.30C(K)\right\}. \tag{S1}$$

The first three terms measure properties of the observed module-size distribution, whereas $C(K)$ is an explicit interpretability prior.

Graph quality. Graph quality evaluates whether the modules form internally cohesive and externally separated communities in G_{DEG}. For a graph $G = (V, E)$, let $m = |E|$, A_{uv} be its adjacency matrix, and d_u be the degree of node u. The modularity of the analyzed partition was calculated as

$$Q_G = \frac{1}{2m}\sum_{u,v\in V}\left(A_{uv} - \frac{d_u d_v}{2m}\right)\mathbb{I}(z_u = z_v),$$

where z_u is the module label of node u. Nodes outside the analyzed modules were assigned separate singleton labels when modularity was calculated. Modularity was transformed to the unit interval as

$$Q_G^* = \text{clip}_{[0,1]}\left(\frac{Q_G + 0.1}{0.9}\right).$$

For module M_i, within-module density was defined as

$$\rho_G(M_i) = \frac{2e_i^{\text{in}}}{s_i(s_i-1)},$$

where e_i^{in} is the number of edges with both endpoints in M_i. Let

$$\text{cut}_G(M_i, \overline{M_i})$$

denote the number of edges between M_i and the remaining graph, and let

$$\text{vol}_G(M_i) = \sum_{v\in M_i} d_v.$$

The normalized boundary conductance used in the analysis was

$$\phi_G(M_i) = \frac{\text{cut}_G(M_i, \overline{M_i})}{\min\{\text{vol}_G(M_i), \text{vol}_G(\overline{M_i})\}}.$$

When the denominator was zero, conductance was set to one. Mean separation was then defined as

$$\text{Sep}_G = 1 - \frac{1}{K}\sum_{i=1}^{K}\phi_G(M_i).$$

A high separation score indicates that relatively few edges leave the modules compared with the graph volume on either side of their boundaries.

Let $\rho(G)$ denote the global density of graph G. Mean within-module density enrichment over the global density was calculated as

$$\overline{\Delta\rho}_G = \frac{1}{K}\sum_{i=1}^{K}\text{clip}_{[0,1]}\left\{\frac{\rho_G(M_i) - \rho(G)}{1 - \rho(G)}\right\}.$$

This term is high when the identified modules are substantially denser than the network as a whole.

Graph quality was calculated on the DEG-overlap network as

$$S_{\text{graph}} = 100\left\{0.40Q^*_{G_{\text{DEG}}} + 0.30\,\text{Sep}_{G_{\text{DEG}}} + 0.30\overline{\Delta\rho}_{G_{\text{DEG}}}\right\}. \tag{S2}$$

The three terms capture complementary aspects of community structure: global modular organization, boundary separation, and local density enrichment.

Background-network cohesion. Graph quality describes the partition within the DEG-overlap network used for module detection. Background-network cohesion instead asks whether the same DEG modules remain cohesive and separated after they are projected onto the full background network.

The score was calculated by applying exactly the same structural formula to G_{BG}:

$$S_{\text{BG}} = 100 \left\{ 0.40 Q^{*}_{G_{\text{BG}}} + 0.30 \, \text{Sep}_{G_{\text{BG}}} + 0.30 \overline{\Delta\rho}_{G_{\text{BG}}} \right\}. \tag{S3}$$

Internal density was evaluated using background-network edges between genes in the same module, whereas conductance included edges from module genes to all other genes in the background network. Consequently, this score measures whether modules identified from the DEG-overlap graph preserve recognizable topological boundaries in the broader interaction or co-expression network.

Background-network cohesion is a descriptive property of individual modules and their boundaries. It does not include MoBC inter-module distance, RandSDM significance, significantly close module pairs, or key-link gene results.

Enrichment consistency. Enrichment consistency evaluates whether the modules correspond to statistically supported and non-redundant biological functions. KEGG pathway and Gene Ontology Biological Process over-representation analyses were performed separately for each module using the corresponding network-specific Entrez-gene universe. Enrichment p-values were adjusted for multiple testing, and terms with BH-adjusted $p < 0.01$ were regarded as significant.

For annotation branch $b \in \{\text{KEGG}, \text{GO}\}$, let $T_{i,b}$ be the set of significant terms for module M_i. Enrichment presence was defined as

$$P_b = \frac{1}{K} \sum_{i=1}^{K} \mathbb{I}(|T_{i,b}| > 0).$$

This term measures the proportion of modules with at least one statistically supported functional annotation.

Let $q_{i,b,t}$ be the BH-adjusted enrichment p-value for term t in module i. Module-level enrichment strength was defined as

$$h_{i,b} = \begin{cases} \min \left\{ 1, \dfrac{-\log_{10}\left(\min_{t \in T_{i,b}} q_{i,b,t}\right)}{10} \right\}, & |T_{i,b}| > 0, \\ 0, & |T_{i,b}| = 0, \end{cases}$$

and the mean branch-specific strength was

$$H_b = \frac{1}{K} \sum_{i=1}^{K} h_{i,b}.$$

Thus, smaller adjusted p-values increase the score, with the contribution capped at one.

For modules with significant enrichment, functional distinctness was quantified using pairwise Jaccard dissimilarity between enriched-term sets:

$$J(T_{i,b}, T_{j,b}) = \frac{|T_{i,b} \cap T_{j,b}|}{|T_{i,b} \cup T_{j,b}|}.$$

When at least two modules had significant terms,

$$R_b = \frac{2}{K_b^*(K_b^* - 1)} \sum_{i<j} \left\{ 1 - J(T_{i,b}, T_{j,b}) \right\},$$

where K_b^* is the number of enriched modules. Following the implemented scoring rule, R_b was set to 0 when no module was enriched and to 0.5 when only one module was enriched. A high value indicates that different modules are associated with distinct functional terms rather than repeatedly recovering the same annotations.

The branch-specific score was

$$S_b = 100\,(0.30P_b + 0.40H_b + 0.30R_b)\,,$$

and the final enrichment-consistency score was

$$S_{\text{enrich}} = \frac{S_{\text{KEGG}} + S_{\text{GO}}}{2}. \tag{S4}$$

Accordingly, enrichment consistency rewards broad functional interpretability, strong enrichment evidence, and functional differentiation among modules. It does not measure direct agreement between KEGG and GO term identities, rather, it combines parallel evaluations from the two annotation systems.

Stability across adjacent parameter values. Stability measures local robustness to the method-specific tuning parameter. It asks whether a small movement to the neighboring tested parameter value produces a similar collection of modules.

For two module collections $\mathcal{M} = \{M_1, \ldots, M_K\}$ and $\mathcal{M}' = \{M'_1, \ldots, M'_{K'}\}$, the directional best-match similarity was

$$BM(\mathcal{M} \to \mathcal{M}') = \frac{1}{K}\sum_{i=1}^{K} \max_{1\le j\le K'} J(M_i, M'_j).$$

Because the number and sizes of modules may differ between settings, the directional similarities were averaged:

$$S(\mathcal{M}, \mathcal{M}') = \frac{1}{2}\left\{BM(\mathcal{M} \to \mathcal{M}') + BM(\mathcal{M}' \to \mathcal{M})\right\}.$$

This bidirectional matching makes the comparison invariant to arbitrary module labels and reduces asymmetry when a module splits or merges.

Let $\mathcal{N}(\theta)$ denote the immediately adjacent values in the tested parameter grid for the same method. Interior values were compared with the preceding and following values, whereas parameter-grid endpoints were compared with their single neighbor. Stability was defined as

$$S_{\text{stability}}(\theta) = 100\frac{1}{|\mathcal{N}(\theta)|}\sum_{\phi\in\mathcal{N}(\theta)} S\left(\mathcal{M}^{(\theta)}, \mathcal{M}^{(\phi)}\right). \tag{S5}$$

Neighboring Walktrap steps, Leiden and Louvain resolutions, and Infomap Markov times were evaluated in this manner. The score therefore represents robustness across the tested grid and should not be interpreted as change per unit increase in the numerical parameter.

Reproducibility under data and seed perturbations. Reproducibility measures whether the module structure is preserved when the input DEG threshold or random seed is perturbed while the method and its tuning parameter are held fixed.

Let $\mathcal{M}^{(\theta,0)}$ be the modules obtained using the primary DEG cutoff. Let $\mathcal{M}^{(\theta,\text{low})}$, $\mathcal{M}^{(\theta,\text{high})}$, and $\mathcal{M}^{(\theta,\text{seed})}$ denote the modules obtained using a lower DEG cutoff, a higher DEG cutoff, and an alternative random seed, respectively. The similarities were

$$R_{\text{low}}(\theta) = S\left(\mathcal{M}^{(\theta,0)}, \mathcal{M}^{(\theta,\text{low})}\right),$$

$$R_{\text{high}}(\theta) = S\left(\mathcal{M}^{(\theta,0)}, \mathcal{M}^{(\theta,\text{high})}\right),$$

and

$$R_{\text{seed}}(\theta) = S\left(\mathcal{M}^{(\theta,0)}, \mathcal{M}^{(\theta,\text{seed})}\right).$$

The final reproducibility score was

$$S_{\text{reproducibility}}(\theta) = \frac{100}{3}\left\{R_{\text{low}}(\theta) + R_{\text{high}}(\theta) + R_{\text{seed}}(\theta)\right\}. \tag{S6}$$

For BRCA-PPI and BRCA-WGCN, the primary threshold was $|\log_2 \text{FC}| > 2.0$ with adjusted $p < 0.01$, and the lower and higher fold-change cutoffs were 1.8 and 2.2. For CAC-PPI, the primary threshold was $|\log_2 \text{FC}| > 1.0$ with adjusted $p < 0.05$, and the corresponding perturbation cutoffs were 0.8 and 1.2. The adjusted-p-value cutoff was held constant during fold-change perturbation.

Stability and reproducibility therefore quantify different forms of robustness. Stability varies the method-specific tuning parameter while keeping the data fixed, whereas reproducibility varies the input DEG set or random seed while keeping the tuning parameter fixed.

The complete trajectories of all six component scores are shown for each method in Figures S3–S6. These figures report the component scores separately and do not incorporate downstream module-pair distances, RandSDM significance, or key-link gene results.

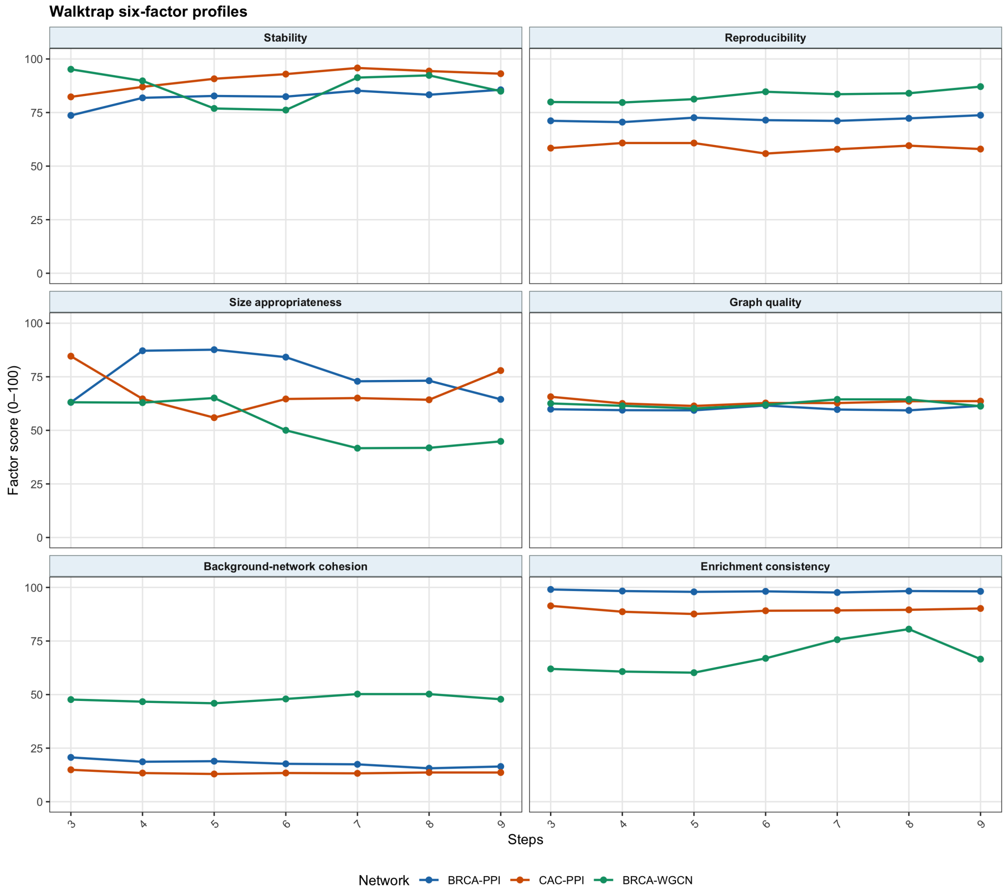

Fig. S3. Walktrap component-score profiles across random-walk steps 3–9. Each panel shows one component score on a 0–100 scale, with higher values indicating a more favorable result. Colored trajectories represent the three analyzed networks.

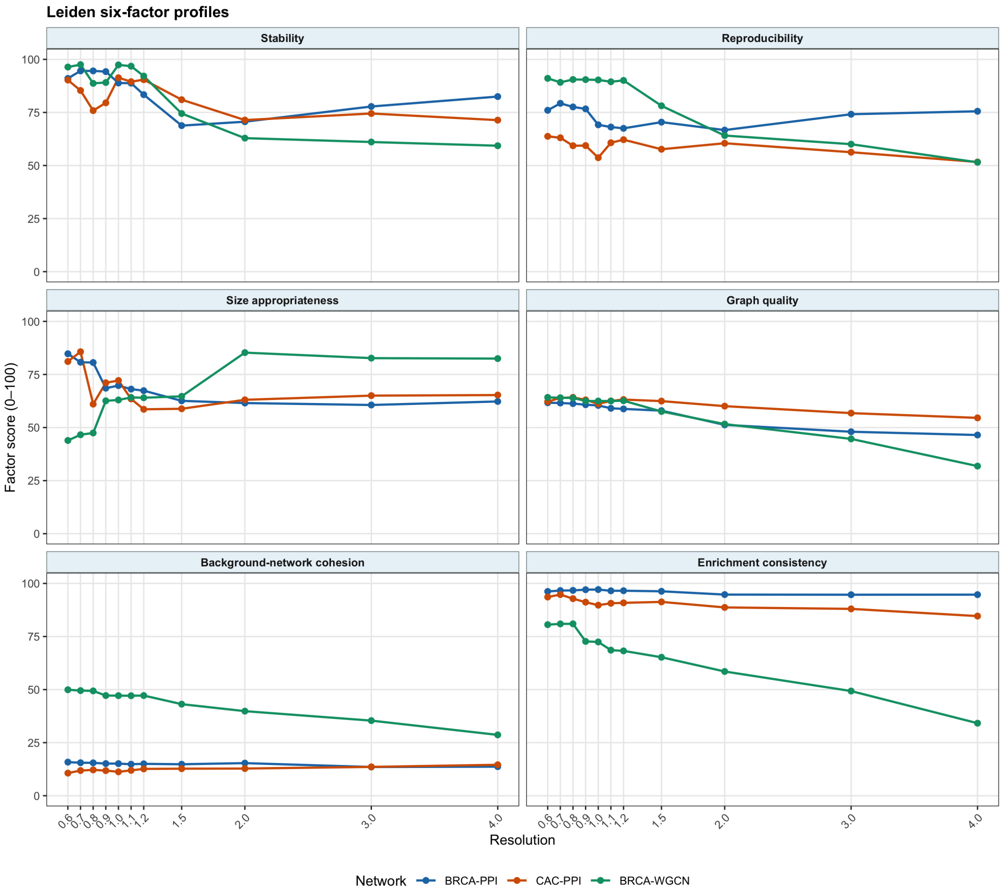

Fig. S4. Leiden component-score profiles across resolution values 0.6–4.0. Each panel shows one component score on a 0–100 scale, with higher values indicating a more favorable result. Colored trajectories represent the three analyzed networks.

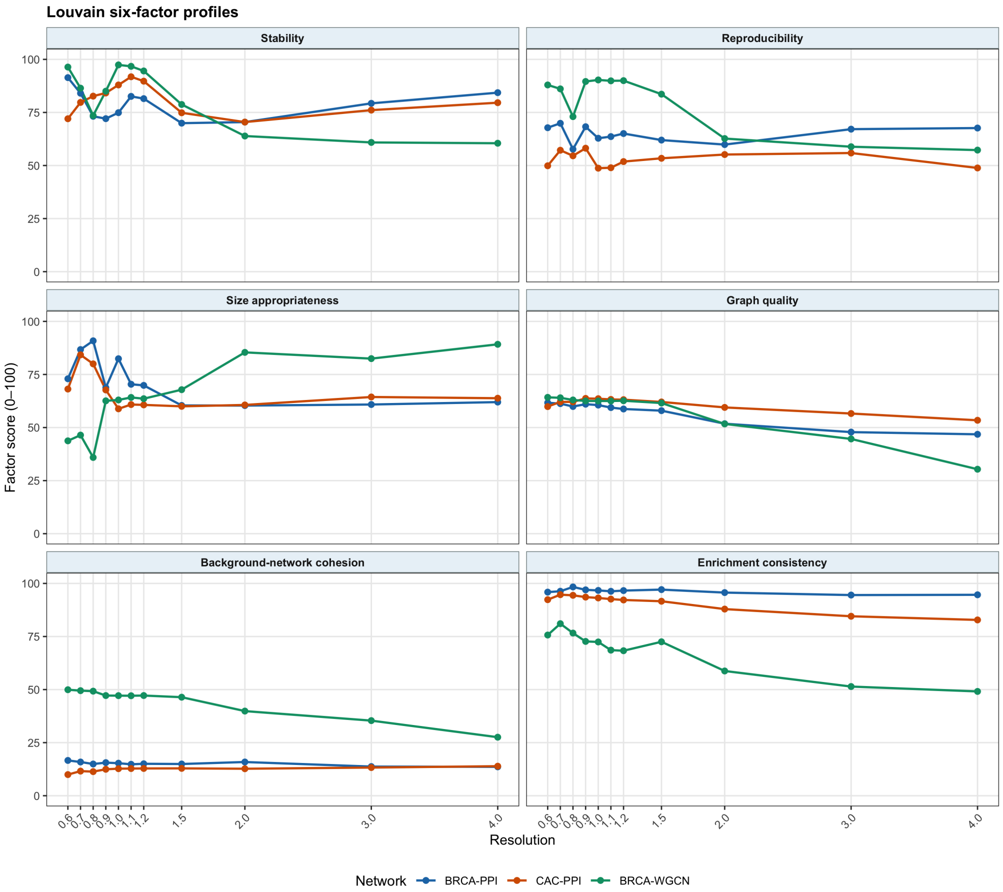

Fig. S5. Louvain component-score profiles across resolution values 0.6–4.0. Each panel shows one component score on a 0–100 scale, with higher values indicating a more favorable result. Colored trajectories represent the three analyzed networks.

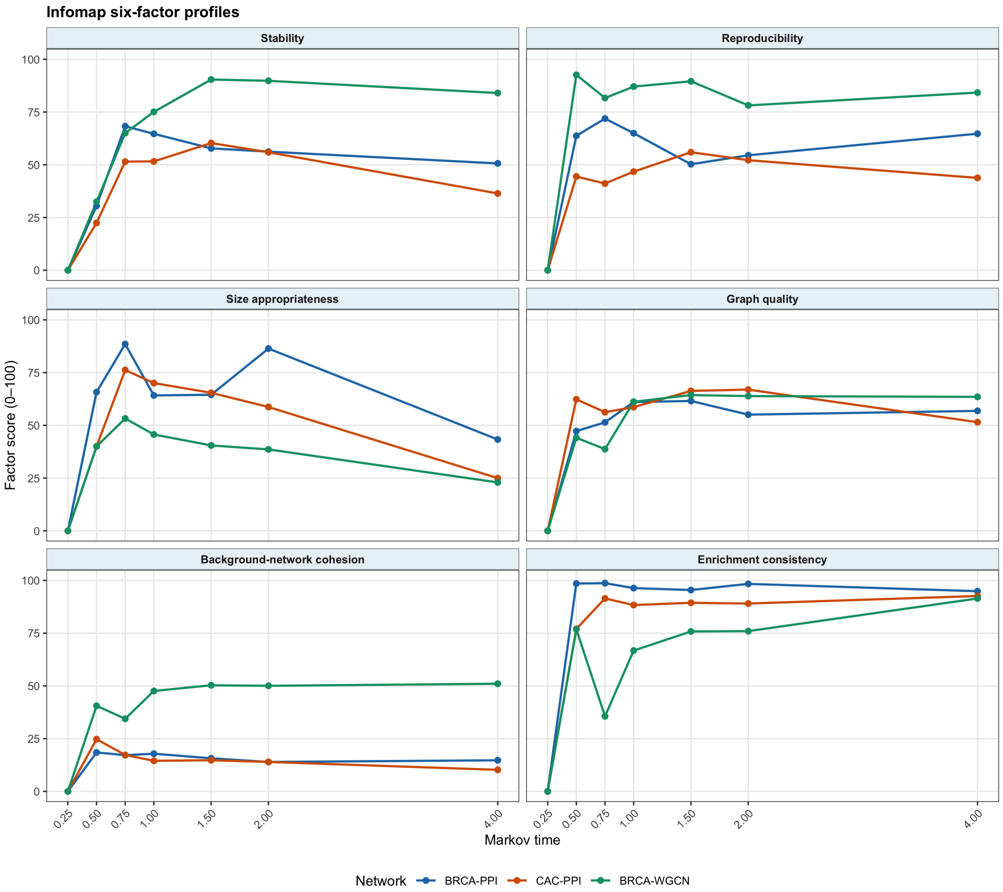

Fig. S6. Infomap component-score profiles across Markov times 0.25–4.0. Each panel shows one component score on a 0–100 scale, with higher values indicating a more favorable result. Colored trajectories represent the three analyzed networks.

C. Factor dependency

Pooled Spearman analysis across 108 method-parameter settings identified the strongest positive association between reproducibility and background-network cohesion ($\rho = 0.764$), followed by stability and graph quality ($\rho = 0.634$). Stability and reproducibility were also correlated ($\rho = 0.507$). The first two standardized principal components explained 81.32% of the six-component variation (52.70% and 28.61%; Figure S7). The components were therefore not treated as conditionally independent evidence.

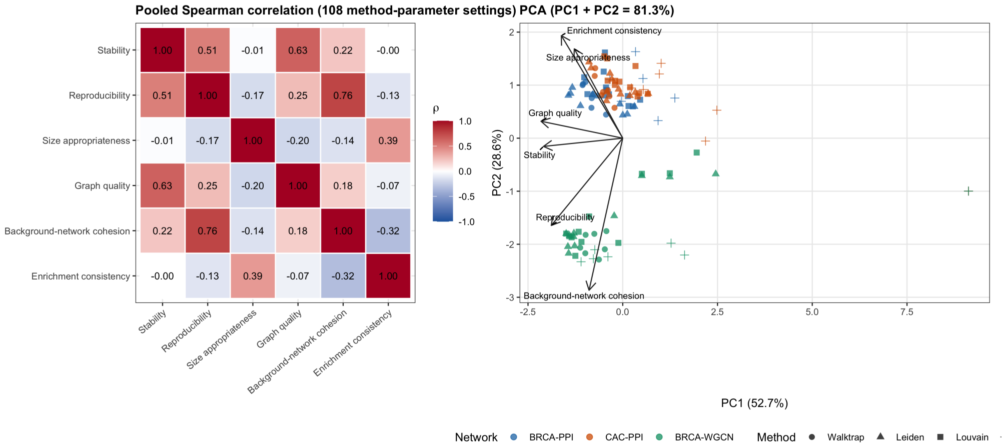

Fig. S7. Dependency among the six component scores. Left: pooled Spearman correlations. Right: standardized PCA. Colors indicate networks and shapes indicate methods.

D. Domain-balanced selection score, eligibility, and practical equivalence

To avoid double-counting correlated factors as six independent criteria, the six factors were grouped into four conceptual domains representing robustness (R), partition quality (P), background-network cohesion (M), and biological coherence (B):

$$R = \frac{S_{\text{stability}} + S_{\text{reproducibility}}}{2}, \qquad P = \frac{S_{\text{size}} + S_{\text{graph}}}{2},$$
$$M = S_{\text{BG}}, \qquad B = S_{\text{enrich}}.$$

The domain-balanced selection score is

$$S_{\text{selection}} = 0.25R + 0.25P + 0.25M + 0.25B. \tag{S7}$$

All inputs were already on a 0–100 scale. Domain weights were fixed rather than fitted to downstream performance. Equal weights were assigned to the four conceptual domains. MoBC, S2B, FCS, RandSDM, GO-distance correlation, and key-link results were not used to select parameters.

Only settings with at least five modules passing the network-specific minimum-size filter ($K_{\text{analyzed}} \geq 5$) were eligible. The parameter with the maximum $S_{\text{selection}}$ was selected within each network–method combination. When a lower-complexity setting lay within one score point of the maximum, it was treated as practically equivalent and chosen for parsimony. This rule selected BRCA-PPI Walktrap steps 4 rather than 5 and Leiden resolution 0.6 rather than 0.7. Figure S8 displays every tested setting and distinguishes settings that did and did not satisfy this minimum module-count criterion.

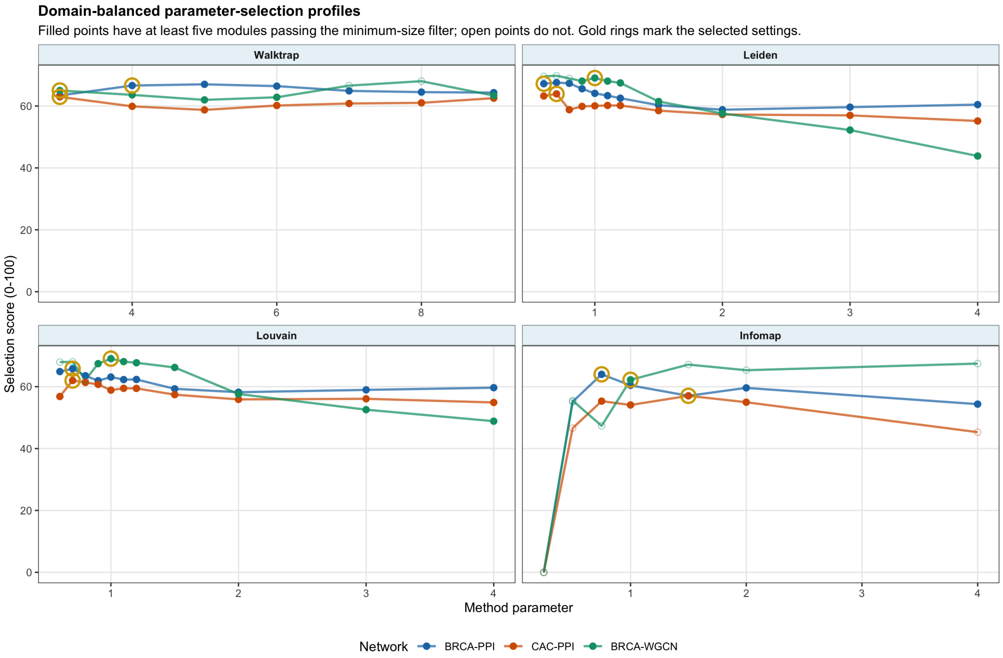

Fig. S8. Domain-balanced parameter-selection profiles. Filled colored points have at least five modules passing the minimum-size filter, open points do not, and gold rings mark selected settings. All tested values are shown so that the BRCA-WGCN trajectory is visible in every panel. In the Infomap panel, only the green point at Markov time 1.0 is eligible for BRCA-WGCN, and the other green points are shown as open circles.

Table S4. Selected parameter settings, domain-balanced selection scores, analyzed-module counts, and module sizes.

Network	Method	Selected parameter	Score	K	Module sizes (descending)	Selection basis
BRCA-PPI	Walktrap	steps 4	66.612	8	164,81,42,41,33,27,25,21	Within 1 point of steps 5; parsimonious choice
BRCA-PPI	Leiden	resolution 0.6	67.196	8	279,156,153,128,89,55,25,24	Within 1 point of 0.7; parsimonious choice
BRCA-PPI	Louvain	resolution 0.7	65.790	8	324,154,121,92,88,56,43,25	Score maximum
BRCA-PPI	Infomap	Markov time 0.75	63.984	8	47,47,40,34,24,23,23,21	Score maximum
CAC-PPI	Walktrap	steps 3	62.956	9	92,75,44,17,15,13,13,13,12	Score maximum
CAC-PPI	Leiden	resolution 0.7	63.915	9	108,95,59,49,43,33,21,15,13	Score maximum
CAC-PPI	Louvain	resolution 0.7	61.981	9	107,72,71,53,34,33,24,15,13	Score maximum
CAC-PPI	Infomap	Markov time 1.5	57.038	11	145,42,21,20,17,16,15,14,14,13,11	Score maximum
BRCA-WGCN	Walktrap	steps 3	65.016	6	183,176,152,74,43,33	Maximum among eligible settings
BRCA-WGCN	Leiden	resolution 1.0	69.046	5	221,191,158,121,40	Maximum among eligible settings
BRCA-WGCN	Louvain	resolution 1.0	69.046	5	221,191,158,121,40	Score maximum
BRCA-WGCN	Infomap	Markov time 1.0	62.211	5	379,155,100,41,40	Only eligible setting

E. Four-domain weight sensitivity

To evaluate sensitivity to the four domain weights, each weight was varied from 0.10 to 0.40 in increments of 0.05 while the four weights were constrained to sum to one. This produced 231 admissible weight sets. For each weight set, the highest-scoring parameter was recorded separately for every network and detection method. Thus, a frequency of 100% means that the same parameter maximized the score under all 231 weight sets, whereas a split indicates weight-sensitive ranking. Infomap 0.75 in BRCA-PPI and Infomap 1.0 in BRCA-WGCN were selected in 100% of weight sets. CAC-PPI Walktrap was less stable, with steps 3 selected in 66.2% and steps 9 in 33.8%. Figure S9 reports score maxima only. The one-point practical-equivalence choices for BRCA-PPI Walktrap and Leiden are applied after this sensitivity calculation.

For BRCA-PPI Walktrap, steps 5 yielded the numerical maximum of the domain-balanced selection score (67.007), whereas steps 4 yielded a score of 66.612 ($\Delta = 0.395$). Under all 231

admissible domain-weight sets, steps 4 remained within the prespecified one-point practical-equivalence margin of the maximum. We therefore treated steps 4 and 5 as practically equivalent and selected the smaller walk step.

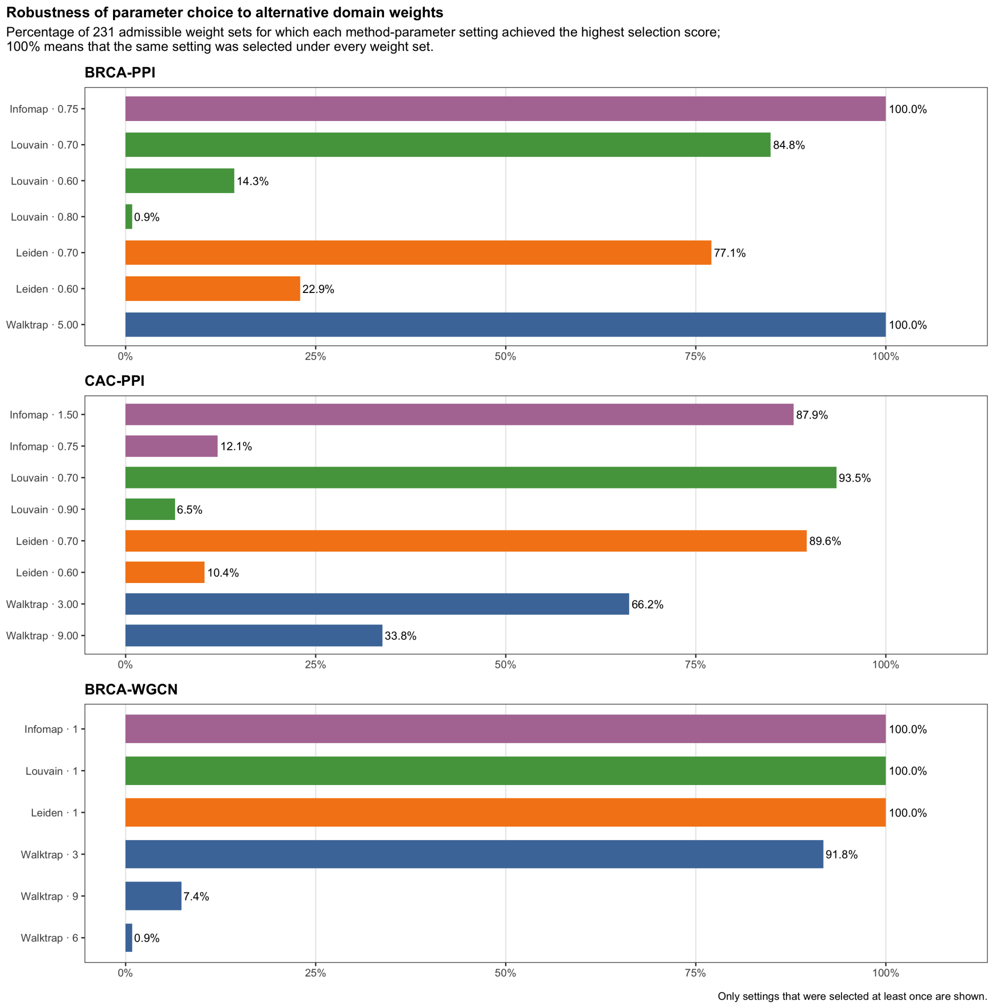

Fig. S9. Sensitivity of the top-scoring parameter to alternative domain weights. The four domains were robustness, partition quality, background-network cohesion, and enrichment consistency. Each domain weight was varied from 0.10 to 0.40 in increments of 0.05, subject to the constraint that the four weights summed to one, resulting in 231 weight sets. For each network and community-detection method separately, we recorded the parameter value with the highest weighted score under each weight set. Bar length represents the percentage of the 231 weight sets for which the indicated parameter was top-scoring. Percentages within each network–method combination therefore sum to 100%. Parameters that were never top-scoring are not displayed. A 100% bar indicates that the top-scoring parameter was unchanged throughout the examined weight grid. Colors identify community-detection methods and do not represent comparisons between methods. The analysis reports score maxima and does not incorporate the subsequent practical-equivalence rule.

4. S2B AND FCS DEFINITIONS AND COMPUTATIONAL COMPLEXITY

A. S2B and FCS

S2B restricts cross-module endpoint pairs to those whose shortest-path distance does not exceed the background-network mean shortest-path length $\bar{d}$ [2]. Let

$$\mathcal{S}_{UW} = \{(u, w) \in U \times W : d_{uw} \leq \bar{d}\}$$

and

$$\mathcal{S}_{UW}(v) = \{(u,w) \in U \times W : v \in SP_{\bar{d}}(u,w)\},$$

where $SP_{\bar{d}}(u,w)$ is the set of shortest paths between u and w whose lengths are at most $\bar{d}$. Then

$$D_{\text{S2B}}(U,W) = \frac{1}{|\mathcal{S}_{UW}|} \sum_{(u,w)\in\mathcal{S}_{UW}} d_{uw}, \tag{S8}$$

with the graph mean used as a fallback if $\mathcal{S}_{UW}$ is empty. The corresponding centrality is the fraction of these eligible shortest-path endpoint pairs whose shortest paths contain v such that

$$c_{\text{S2B}}^{UW}(v) = \frac{|\mathcal{S}_{UW}(v)|}{|\mathcal{S}_{UW}|}. \tag{S9}$$

FCS treats all shortest paths between U and W as flow [5]:

$$c_{\text{FCS}}^{UW}(v) = \frac{1}{|U||W|} \sum_{u\in U, w\in W} \frac{\sigma_{uw}(v)}{\sigma_{uw}}, \tag{S10}$$

$$D_{\text{FCS}}(U,W) = \frac{1}{|U||W|} \sum_{u\in U, w\in W} d_{uw}. \tag{S11}$$

Because G is undirected, both S2B and FCS distances are symmetric. Their link-gene scores are also invariant to swapping U and W.

B. Computational complexity

Let $n = |V|$, $m = |E|$, module sizes be s_i, s_j, K be the number of analyzed modules, and R be the number of randomizations. Repeated breadth-first search for all-pairs distances costs $O(n(n+m))$ time and $O(n^2)$ memory. Once distances are cached, a distance for one module pair costs $O(s_i s_j)$ and all observed pairs cost $O(\sum_{i<j} s_i s_j)$. RandSDM normalization costs approximately

$$O\left(n(n+m) + R\sum_{i<j} s_i s_j\right),$$

excluding cached sampling and input/output.

Key-link analysis additionally enumerates shortest paths. If $\Pi_{ij}^{\text{nearest}}$ is the number of enumerated nearest-endpoint shortest paths, observed MoBC key-link calculation is approximately $O((s_i + s_j)(n+m) + \Pi_{ij}^{\text{nearest}})$. S2B and FCS can enumerate many more endpoint pairs. Runtime profiling showed that one randomization for TCGA-BRCA M2–M3 required 11.28 seconds for MoBC, 46.25 seconds for FCS, and 49.06 seconds for S2B; CAC M4–M8 required 2.19, 5.11, and 13.31 seconds, respectively (Table S5). To limit computation, gene-specific randomization is performed only for significantly closest MoBC pairs, and online sufficient statistics are stored instead of complete null matrices.

Table S5. Runtime comparison for one randomization iteration in key link gene analysis. Runtime is reported in seconds for representative significant module pairs from the TCGA-BRCA and CAC networks.

Method	TCGA-BRCA M2–M3	CAC M1–M2	CAC M4–M8
MoBC	11.27534	12.45841	2.18617
FCS	46.25118	86.13858	5.10502
S2B	49.05750	141.49230	13.31436

5. ABIN, RANDSDM, ADAPTIVE STOPPING RULE, AND MULTIPLE-TESTING SCOPES

A. ABin and RandSDM

ABin adaptively merges degree bins to balance degree preservation and sampling coverage, whereas TBin uses fixed-size degree bins [3]. RandSD draws size- and degree-matched random

modules. RandSDM samples candidates sequentially with distance-weighted probabilities favoring nodes near the partially sampled set, thereby tending to preserve local compactness and modularity. This is a stochastic tendency, not a guarantee that every RandSDM draw has higher modularity than every RandSD draw.

The parameter scan supported ABin $\kappa = 0.07$ and the reference TBin threshold $\tau = 100$. ABin $\kappa = 0.07$ avoided unsampled bins while providing a maximum node-sampling count and degree-distribution difference comparable with TBin $\tau = 100$. RandSDM produced higher modularity and diameters closer to the observed disease gene modules than RandSD (Figures S10 and S11). ABin $\kappa = 0.07$ was therefore used throughout the parameter-grid analysis.

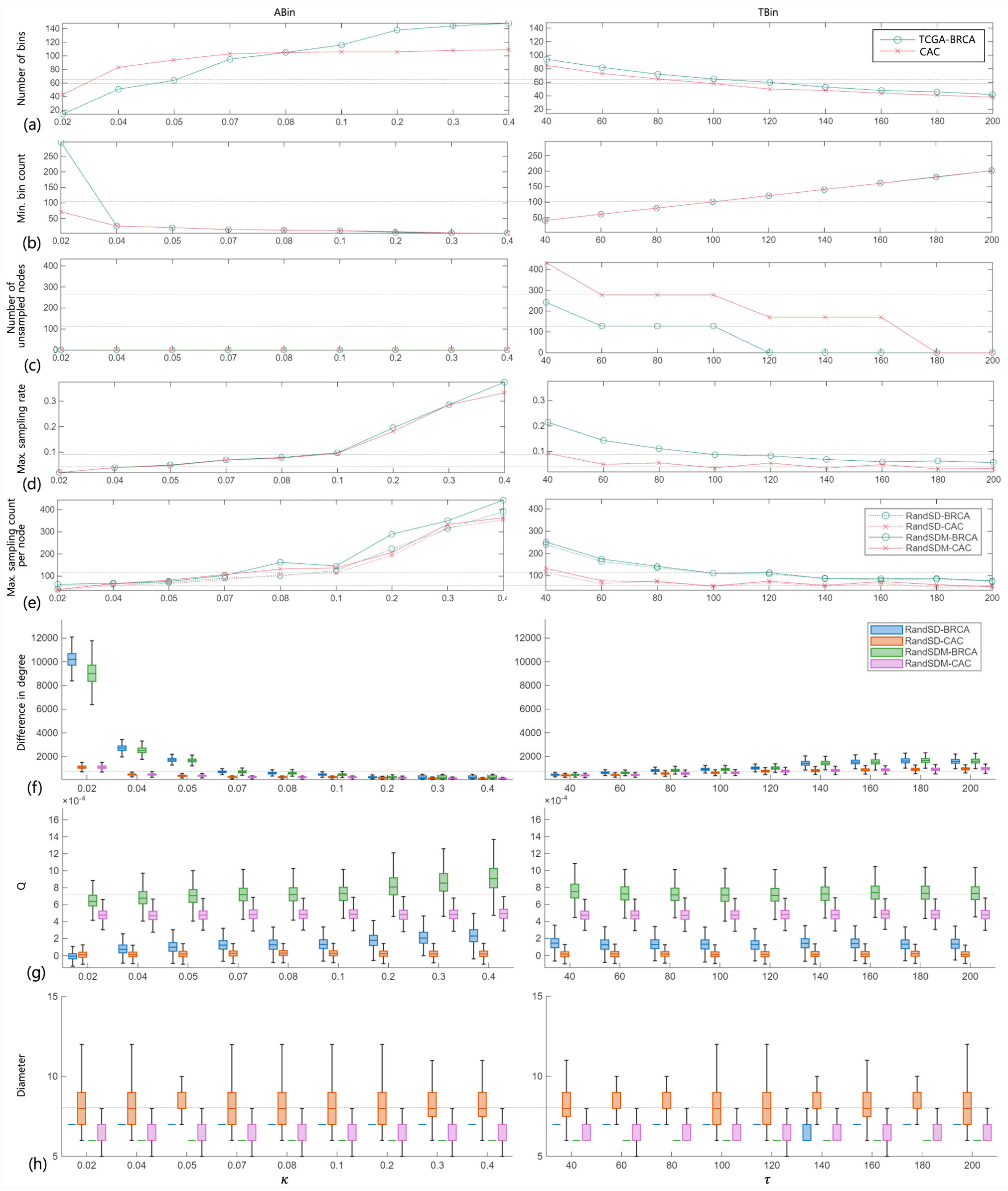

Fig. S10. ABin/TBin parameter comparison. Panels summarise bin count, minimum bin size, unsampled nodes, maximum sampling probability/count, degree distortion, modularity, and diameter.

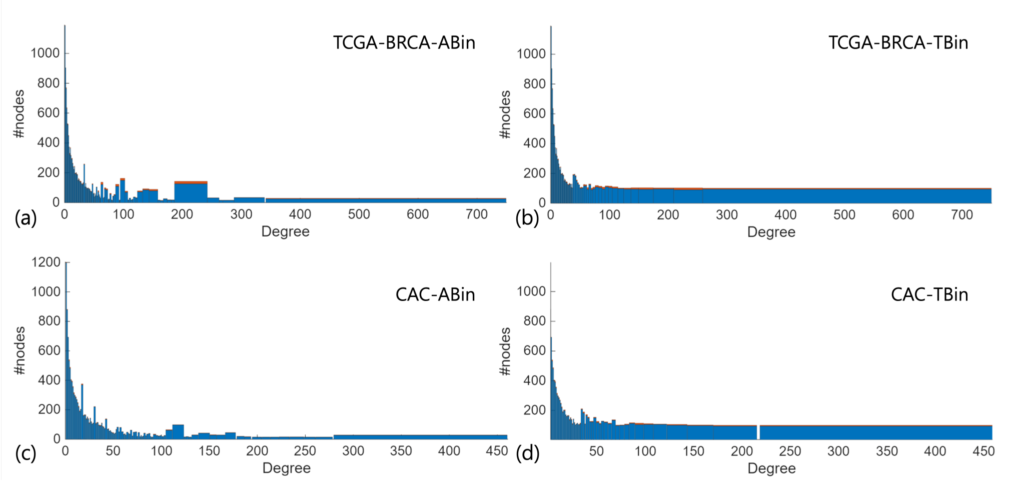

Fig. S11. Binned degree distributions for ABin $\kappa = 0.07$ and TBin $\tau = 100$ in BRCA-PPI and CAC-PPI.

B. Shared random modules for MoBC, S2B, and FCS

For each method-parameter setting, one random module pair (U_b, W_b) was generated at each RandSDM iteration and reused for all three distance measures. For observed distance D_{obs}, the one-sided lower-tail empirical value was

$$p_D = \frac{1 + \sum_{b=1}^{B} \mathbf{1}\{D_b \leq D_{\text{obs}}\}}{B+1}. \tag{S12}$$

Reusing the random pair reduces sampling overhead and makes the three null comparisons paired at the randomization level.

C. Adaptive stopping rule

Distance randomization began at 1,000 iterations. After the initial 1,000 randomizations, each module partition was evaluated in additional batches of 250 randomizations until the stopping criterion was met or the maximum of 5,000 randomizations was reached. After every batch, the algorithm recalculated all empirical P values, $q_{\text{within-setting}}$, and $q_{\text{across-grid}}$. A check was stable only when: (i) the maximum absolute change in any empirical P value was at most 0.01; (ii) among tests with current $P \leq 0.10$, the largest Monte Carlo standard error (MCSE) $\sqrt{p(1-p)/B}$ was at most 0.005; and (iii) the significant-test sets and the significantly closest-pair signatures were unchanged for both correction scopes. Three consecutive stable checks were required. Full batches were used so that provisional $q_{\text{across-grid}}$ values were never compared between incomplete grids.

Across 36 settings per network, adaptive convergence was reached for 27 BRCA-PPI, 25 CAC-PPI, and 30 BRCA-WGCN settings; 8, 9, and 4 settings, respectively, reached 5,000 iterations. One BRCA-PPI setting and two settings in each of CAC-PPI and BRCA-WGCN had fewer than two modules passing the minimum-size filter and were not randomized (Figure S12).

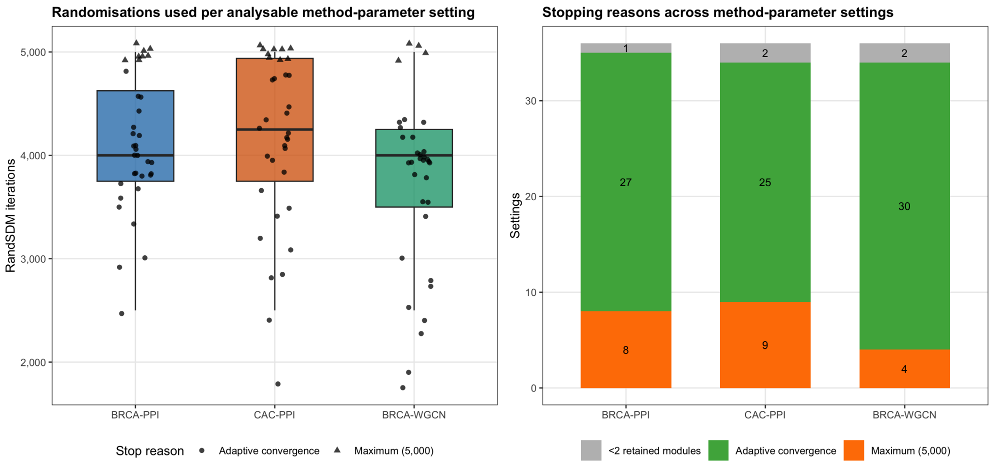

Fig. S12. Adaptive RandSDM. Left: randomizations used among analyzable method-parameter settings. Right: stop reasons across 36 settings per network.

D. Multiple-testing scopes

$q_{\text{within-setting}}$ is BH correction across all module-pair tests for one distance measure within one community-detection method and parameter setting. $q_{\text{across-grid}}$ is BH correction across all module-pair, method, and parameter tests for one distance measure within a network's complete 36-setting grid. Findings at selected settings are reported primarily at $q_{\text{within-setting}} < 0.05$ because parameter selection was performed without using inter-module distances or their significance. Parameter-grid sensitivity findings are interpreted with $q_{\text{across-grid}} < 0.05$ or explicitly labelled exploratory.

For a candidate link gene with observed $c^{UW}_{\text{MoBC}}(v)$, the same fixed gene was evaluated against every randomized module pair. Its randomized score $c_b(v)$ was set to zero when it did not occur on an eligible connecting shortest path or when it was sampled into either randomized module, otherwise its MoBC score for that randomized pair was used. The plus-one upper-tail value was

$$p_v = \frac{1 + \sum_{b=1}^{B} \mathbf{1}\{c_b(v) \geq c_{\text{obs}}(v)\}}{B+1}, \tag{S13}$$

Here, B is the total number of randomized module pairs evaluated for the fixed gene. Gene-level analysis used 1,000–5,000 iterations in batches of 250, the same 0.01 p-change and 0.005 candidate-Monte Carlo standard error (MCSE) thresholds, an unchanged $q_{\text{within-pair}} < 0.05$ gene set, and two consecutive stable checks. Significant genes were ranked by decreasing observed MoBC score, with $q_{\text{within-pair}}$, empirical p, standardized MoBC, and gene symbol used sequentially to resolve score ties. Up to 20 prioritized genes were reported for each selected module pair.

6. GO-BASED BIOLOGICAL CONCORDANCE

A. Biological distances

GO biological-process similarity was calculated by Wang, Resnik, and graph information content (GIC) methods [8, 10, 14, 16]. For terms a, b, Resnik similarity is the information content of their most informative common ancestor. GIC is the information-content sum over common ancestors divided by the sum over the ancestor union. Wang similarity propagates semantic contributions through the GO parent–child DAG. Module-level similarity used best-match average (BMA): the mean of the best term-set match in each direction. Biological distance is $D_{\text{bio}} = 1 - S_{\text{BMA}}$.

B. Weighted correlation and permutation inference

For module pair i, the topological distance was first standardized against its pair-specific RandSDM null distribution:

$$z_{i,\text{topo}} = \frac{D_{i,\text{obs}} - \mu_{i,\text{null}}}{\sigma_{i,\text{null}}}.$$

This is the same topological quantity used for the manuscript analysis. For matching upper-triangle entries $x_i = z_{i,\text{topo}}$ and $y_i = D_{i,\text{bio}}$, weights were $w_i = 1 - p_{i,\text{topo}}$, where $p_{i,\text{topo}}$ is the raw one-sided RandSDM value. Thus, the pair-specific null distribution accounts for differences in module size and network context before the biological and topological distances are compared. The weighted Pearson correlation was

$$r_w = \frac{\sum_i w_i(x_i - \bar{x}_w)(y_i - \bar{y}_w)}{\sqrt{\sum_i w_i(x_i - \bar{x}_w)^2}\sqrt{\sum_i w_i(y_i - \bar{y}_w)^2}}.$$

The weight upweights closer, better-supported module pairs while retaining all finite pairs. Because smaller topological z-scores and smaller biological distances both indicate greater proximity, a positive r_w denotes biological–topological concordance. Two-sided significance was assessed by jointly permuting the row and column labels of the biological matrix. All permutations were enumerated when $K \leq 7$; otherwise 9,999 Monte Carlo permutations were used. We tested the complete 3×3 combination of MoBC/S2B/FCS and Wang/Resnik/GIC in the manuscript. BH values were calculated within each community-detection setting, across the parameter grid of each network, and across all networks, community-detection methods, tested parameter values, and distance combinations.

Across 927 analyzable combinations, 261 had global BH $q < 0.05$. At the 12 selected community-detection settings, 29 combinations remained globally significant (Table S6, Figure S13).

Table S6. Numbers of globally significant biological–topological associations at the selected community-detection settings. Complete correlation, permutation P, and BH-adjusted q values are visualized in Figure S13 and provided in the accompanying result table.

Network	Setting	Selected parameter	Significant	Significant combinations
BRCA-PPI	Walktrap	steps 4	3	MoBC–Wang, MoBC–Resnik, MoBC–GIC
BRCA-PPI	Infomap	Markov time 0.75	3	MoBC–GIC, FCS–Wang, FCS–GIC
CAC-PPI	Leiden	resolution 0.7	2	S2B–Wang, FCS–Wang
CAC-PPI	Louvain	resolution 0.7	4	S2B–Wang, S2B–GIC, FCS–Wang, FCS–GIC
CAC-PPI	Walktrap	steps 3	6	S2B/FCS–Wang, Resnik, GIC
CAC-PPI	Infomap	Markov time 1.5	6	MoBC–Wang/Resnik; S2B/FCS–Wang/Resnik
BRCA-WGCN	Leiden	resolution 1.0	1	FCS–GIC
BRCA-WGCN	Louvain	resolution 1.0	1	FCS–GIC
BRCA-WGCN	Walktrap	steps 3	3	S2B–Resnik/GIC; FCS–GIC

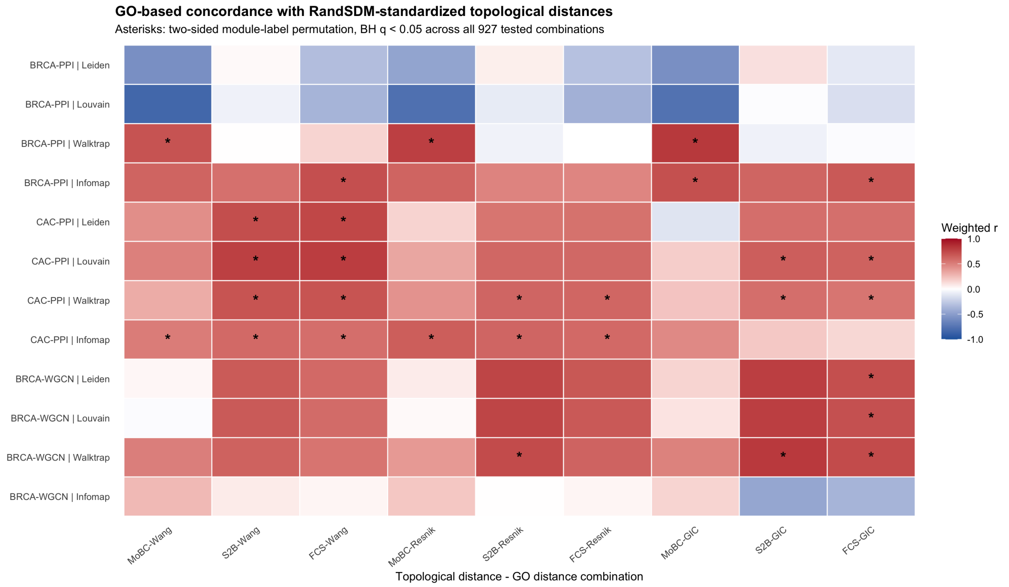

Fig. S13. Weighted correlations between RandSDM-standardized topological distances and GO-based biological distances at the 12 selected community-detection settings. Asterisks indicate two-sided module-label permutation values with global BH $q < 0.05$ across all 927 analyzable network, method, parameter, and 3×3 distance combinations.

For the selected Walktrap partitions, MoBC had the highest mean concordance among the three topological measures in BRCA-PPI (mean $r_w = 0.769$), whereas S2B and FCS were stronger in CAC-PPI and BRCA-WGCN. Across the three networks, Walktrap–MoBC nevertheless had the largest mean weighted correlation of the three Walktrap distance measures (mean $r_w = 0.517$; S2B, 0.438; FCS, 0.434). These results show that concordance depends on both the network and the topological-distance definition and do not imply uniform superiority of one combination in every network.

7. DOWNSTREAM RESULTS AT SELECTED COMMUNITY-DETECTION PARAMETERS

A. Functional enrichment profiles of selected Walktrap modules

The enrichment component was derived from functional profiles of the analyzed modules. Figures S14–S17 show the top five enriched GO biological-process terms and KEGG pathways for each module in the selected Walktrap partitions (BRCA-PPI steps 4, CAC-PPI steps 3). The four panels are presented separately to keep individual terms and module-specific patterns legible. Color intensity represents $-\log_{10}$ of the adjusted enrichment value, white asterisks indicate adjusted $p < 0.01$, and bold labels identify representative terms or pathways.

B. Sensitivity of closest-pair identification to the distance definition

Section 3 selected one community-detection parameter for each network–method combination using the prespecified component scores. This produced 12 selected module sets: four community-detection methods for each of the three networks.

For each selected module set, all pairwise inter-module distances were calculated separately using MoBC, S2B, and FCS. The same randomized module pairs were used to construct the RandSDM null distributions for the three distance measures. For each observed module pair, a one-sided empirical p-value was calculated as the probability of obtaining a randomized distance less than or equal to the observed distance.

Within each network–community-detection method–distance combination, BH correction was applied across the $\binom{K}{2}$ tested module pairs. This is the within-setting correction $q_{\text{within-setting}}$ defined in Section 5. Because 12 module sets and three distance definitions were evaluated, we additionally applied BH correction across all 960 module-pair tests from the selected analyses, denoted by q_{selected}.

A significantly close module pair was defined by

$$q_{\text{within-setting}} < 0.05.$$

When more than one pair satisfied this criterion within a distance matrix, the pair with the smallest observed distance was designated the significantly closest pair for that matrix. If no pair satisfied the criterion, the result was recorded as none. Module numbers correspond to modules ordered by decreasing size.

Table S7. Significantly closest module pairs for the selected module sets. Each distance-method cell reports the significantly closest pair, its within-setting BH-adjusted value $q_{\text{within-setting}}$, and, after the semicolon, the total number of significant pairs for that distance measure. A dash indicates that no pair had $q_{\text{within-setting}} < 0.05$.

Network	Detection method	Selected parameter	MoBC pair (q; n_{sig})	S2B pair (q; n_{sig})	FCS pair (q; n_{sig})
BRCA-PPI	Walktrap	steps 4	M2–M3 (0.00861; 1)	M1–M4 (0.00172; 9)	M1–M4 (0.00108; 10)
BRCA-PPI	Leiden	resolution 0.6	–	M2–M6 (0.00746; 1)	M2–M6 (0.00746; 1)
BRCA-PPI	Louvain	resolution 0.7	–	M2–M6 (0.00560; 4)	M2–M6 (0.00560; 2)
BRCA-PPI	Infomap	Markov time 0.75	M2–M7 (0.00373; 2)	M5–M8 (0.00373; 7)	M5–M8 (0.00187; 11)
CAC-PPI	Walktrap	steps 3	M4–M8 (0.03199; 1)	M4–M8 (0.00200; 8)	M4–M8 (0.00133; 8)
CAC-PPI	Leiden	resolution 0.7	–	M1–M9 (0.00320; 3)	M1–M9 (0.00320; 4)
CAC-PPI	Louvain	resolution 0.7	–	M1–M9 (0.00360; 4)	M1–M9 (0.00360; 4)
CAC-PPI	Infomap	Markov time 1.5	–	M4–M5 (0.00305; 6)	M4–M5 (0.00305; 5)
BRCA-WGCN	Walktrap	steps 3	–	M1–M2 (0.00143; 3)	M1–M2 (0.00143; 5)
BRCA-WGCN	Leiden	resolution 1.0	–	M1–M2 (0.00250; 2)	M1–M2 (0.00125; 3)
BRCA-WGCN	Louvain	resolution 1.0	–	M1–M2 (0.00235; 2)	M1–M2 (0.00235; 3)
BRCA-WGCN	Infomap	Markov time 1.0	–	M3–M4 (0.00083; 3)	M3–M4 (0.00083; 4)

MoBC identified significant pairs in three of the 12 selected module sets: BRCA-PPI Walktrap, BRCA-PPI Infomap, and CAC-PPI Walktrap. BRCA-PPI Infomap contained two significant MoBC

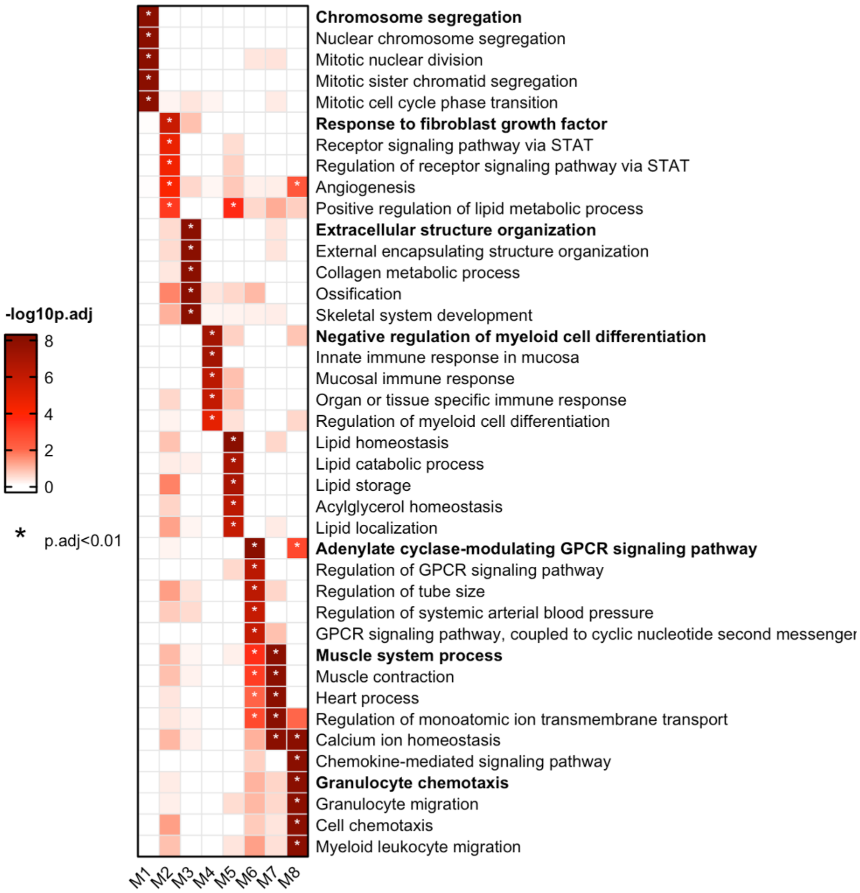

Fig. S14. GO biological-process enrichment of the selected BRCA-PPI Walktrap modules. Rows are the union of the top five terms from each module. Columns are modules ordered by decreasing size.

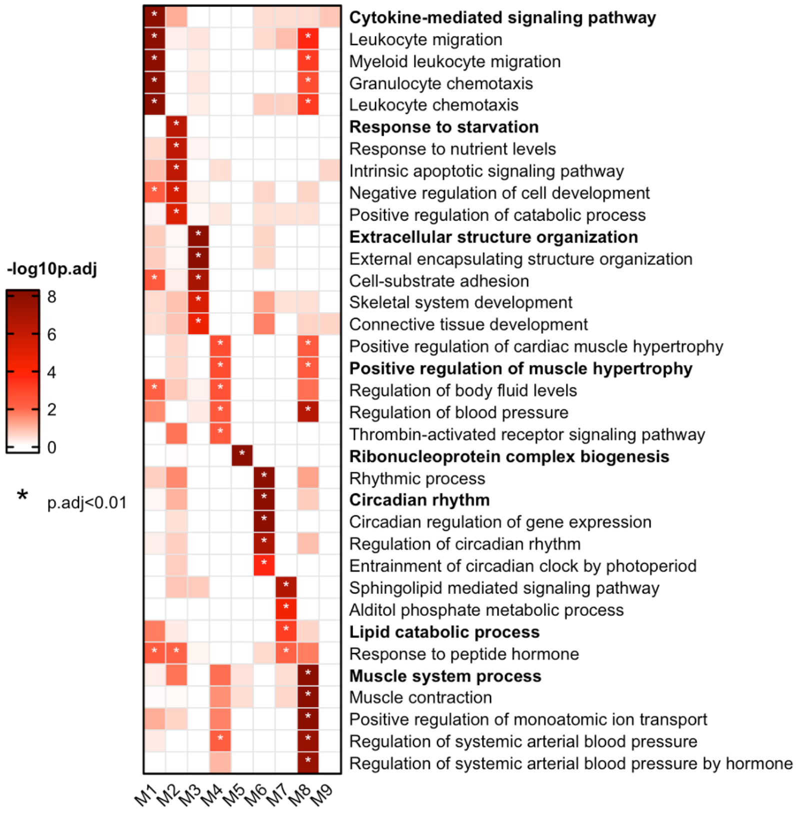

Fig. S15. GO biological-process enrichment of the selected CAC-PPI Walktrap modules. Rows are the union of the top five terms from each module. Columns are modules ordered by decreasing size.

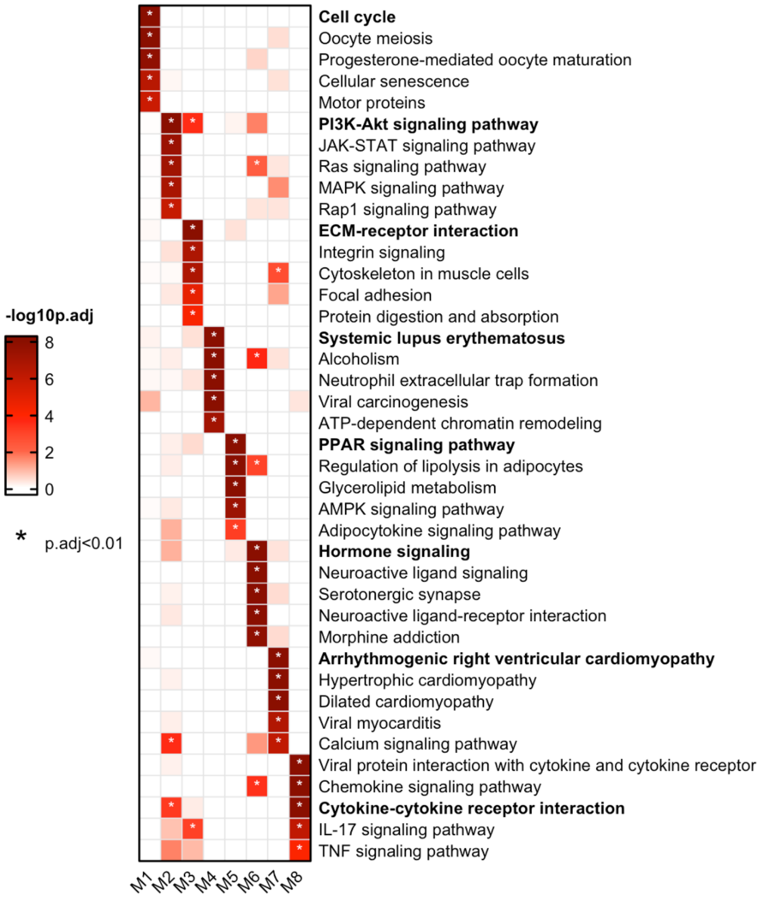

Fig. S16. KEGG pathway enrichment of the selected BRCA-PPI Walktrap modules. Rows are the union of the top five pathways from each module. Columns are modules ordered by decreasing size.

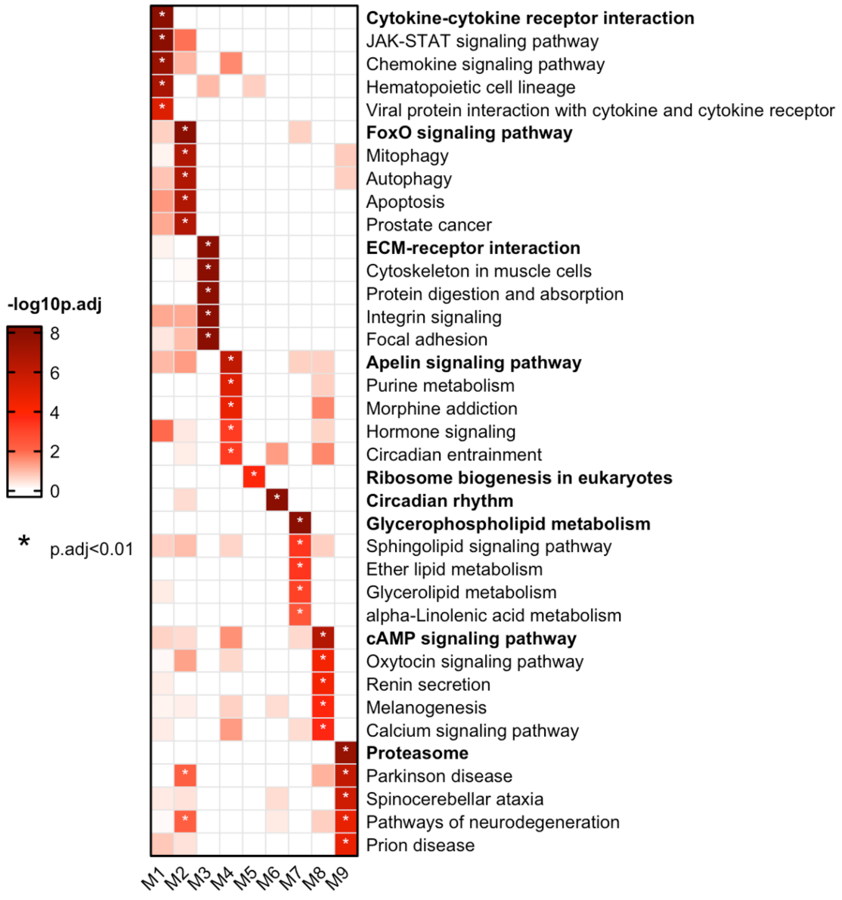

Fig. S17. KEGG pathway enrichment of the selected CAC-PPI Walktrap modules. Rows are the union of the top five pathways from each module. Columns are modules ordered by decreasing size.

pairs; M2–M7 was designated the significantly closest pair because it had the smaller observed MoBC distance. All four reported significant MoBC pair tests remained significant after BH correction across the 960 pairwise tests from the selected analyses ($q_{\text{selected}} \leq 0.0101$).

In contrast, S2B and FCS identified at least one significant pair in every selected module set and generally produced multiple significant pairs. The significantly closest S2B and FCS pair was identical in all 12 module sets. MoBC agreed with S2B and FCS for CAC-PPI Walktrap, where all three measures identified M4–M8, but identified different closest pairs in BRCA-PPI Walktrap and Infomap. These results show that closest-pair identification is sensitive to the distance definition and that MoBC is more selective than S2B and FCS.

Figures S18–S20 show the complete module-pair RandSDM z-score matrices for the three distance measures at the selected parameter settings.

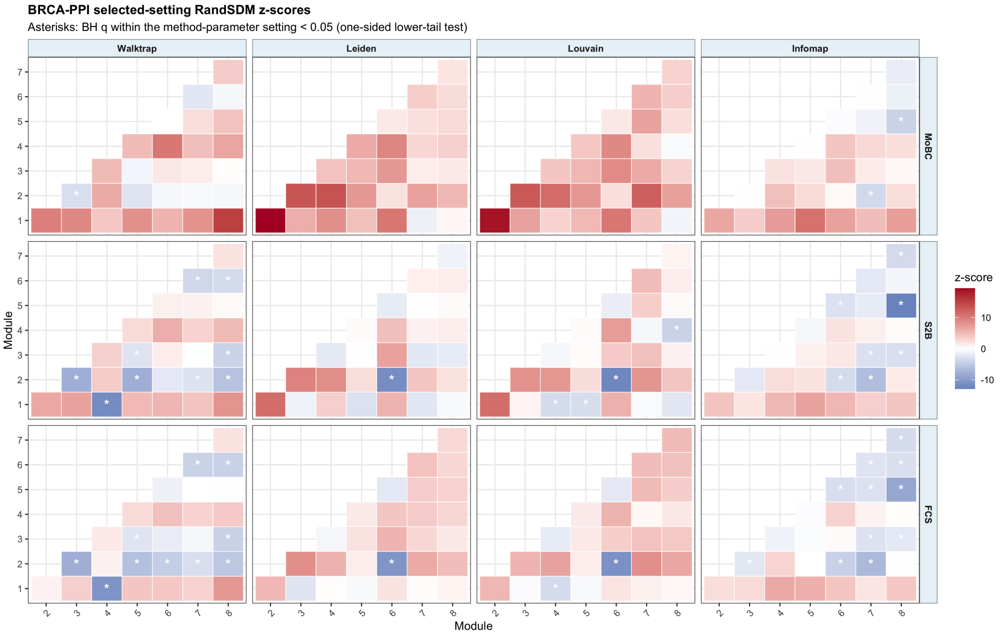

Fig. S18. BRCA-PPI RandSDM z-scores for MoBC, S2B, and FCS using the selected community-detection parameters. Asterisks indicate $q_{\text{within-setting}} < 0.05$.

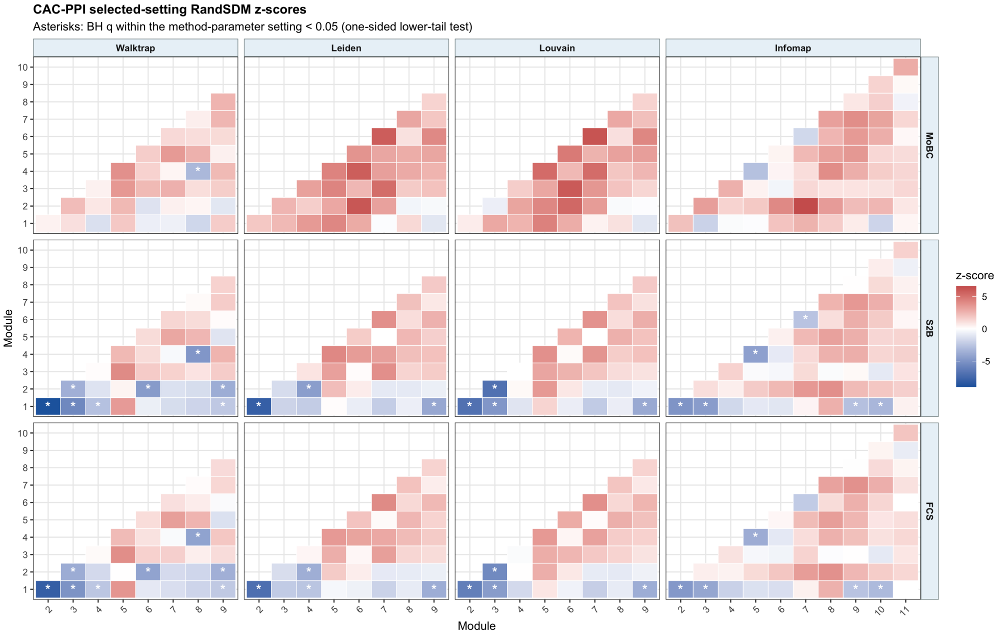

Fig. S19. CAC-PPI RandSDM z-scores for MoBC, S2B, and FCS using the selected community-detection parameters. Asterisks indicate $q_{\text{within-setting}} < 0.05$.

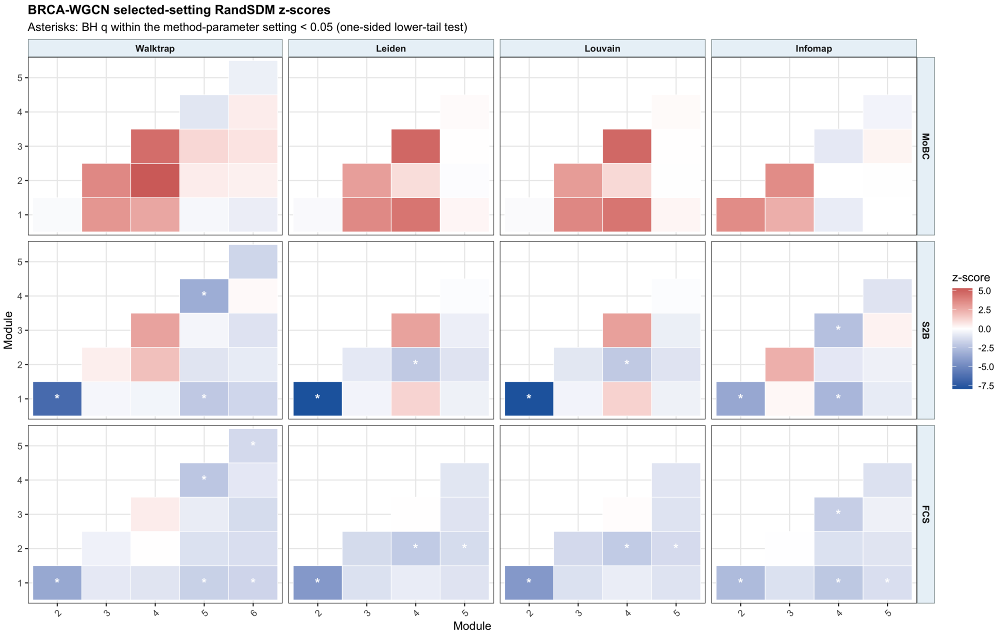

Fig. S20. BRCA-WGCN RandSDM z-scores for MoBC, S2B, and FCS using the selected community-detection parameters. Asterisks indicate $q_{\text{within-setting}} < 0.05$. No selected BRCA-WGCN module set contained a significant MoBC pair.

C. Cross-method consistency of significantly closest MoBC pairs

We compared the significantly closest MoBC pairs obtained from the selected community-detection settings to determine whether the corresponding gene sets remained separated under the other algorithms. Module labels were not compared directly because each method produced a different partition. Instead, each source module was mapped to the target module containing

the largest number of its genes.

In BRCA-PPI, Walktrap identified M2–M3 as the significantly closest MoBC pair ($q_{\text{within-setting}} = 0.00861$), separating a growth-factor and cytokine-signalling module from an extracellular-matrix module. Infomap recovered a related separation as M2–M7 ($q_{\text{within-setting}} = 0.00373$). Infomap M2 contained 41 of the 42 Walktrap M3 genes, whereas Infomap M7 contained 19 genes from Walktrap M2 and represented a finer subset of that module. In contrast, both Walktrap modules mapped predominantly to the same Leiden M1 and Louvain M1. Their relationship was therefore represented as within-module structure rather than an inter-module comparison in those partitions.

In CAC-PPI, Walktrap M4–M8 was the significantly closest MoBC pair ($q_{\text{within-setting}} = 0.03199$), separating cAMP/GPCR-related and calcium/contractile-signalling modules. Leiden placed both modules in M3, and Louvain placed both in M4. Infomap retained the separation almost exactly as M4–M5, but this pair did not remain significant for MoBC after within-setting BH correction ($q_{\text{within-setting}} = 0.110$). Thus, preservation of a similar module separation did not necessarily imply preservation of its RandSDM significance. No selected BRCA-WGCN partition contained a significantly closest MoBC pair.

Table S8. Cross-method comparison of the significantly closest MoBC pairs at the selected community-detection settings. "Same target module" indicates that the two source modules mapped predominantly to one module in the target partition, so their relationship was no longer evaluated as an inter-module pair.

Network	Source pair	Target method	Cross-method relationship
BRCA-PPI	Walktrap M2–M3	Infomap	Recovered as the related M7–M2 separation; Infomap M7 was a finer subset of Walktrap M2, and Infomap M2 closely matched Walktrap M3.
BRCA-PPI	Walktrap M2–M3	Leiden/Louvain	Both source modules mapped predominantly to M1 in each method and were therefore represented as within-module structure.
CAC-PPI	Walktrap M4–M8	Infomap	Preserved as M4–M5, but the MoBC result was not significant after within-setting BH correction ($q_{\text{within-setting}} = 0.110$).
CAC-PPI	Walktrap M4–M8	Leiden/Louvain	Both source modules mapped to Leiden M3 and Louvain M4, respectively, and were therefore not retained as separate inter-module pairs.

D. Gene-specific RandSDM and key-link gene results

Gene-specific RandSDM was restricted to the significantly closest MoBC pair identified at each selected community-detection setting. Three MoBC pairs qualified for gene-level analysis: BRCA-PPI Walktrap M2–M3, BRCA-PPI Infomap M2–M7, and CAC-PPI Walktrap M4–M8. None of the selected BRCA-WGCN partitions contained a significantly closest MoBC pair. Therefore, no BRCA-WGCN key-link gene analysis was performed.

For each qualifying pair, candidate key-link genes were defined as genes located outside both modules and occurring on shortest paths connecting the two modules. Gene-specific RandSDM generated an empirical null distribution for each candidate gene. The resulting empirical p values were adjusted using the BH procedure across all candidate genes tested within the corresponding module pair.

Results were classified using two within-pair FDR tiers. Genes with $q_{\text{within-pair}} < 0.05$ were considered statistically significant under the primary criterion. Because the gene-level analysis was also used as a prioritization screen, genes with $0.05 \leq q_{\text{within-pair}} < 0.07$ were additionally retained as borderline candidates under an expanded screening threshold. These borderline candidates were reported separately and were not interpreted as meeting the primary 5% FDR criterion.

The analysis evaluated 264, 126, and 52 candidate key-link genes for BRCA-PPI Walktrap M2–M3, BRCA-PPI Infomap M2–M7, and CAC-PPI Walktrap M4–M8, respectively. Under the primary criterion, 24, 48, and 36 genes had $q_{\text{within-pair}} < 0.05$. An additional 32, 15, and 2 genes, respectively, had $0.05 \leq q_{\text{within-pair}} < 0.07$, resulting in 56, 63, and 38 genes under the expanded threshold. The adaptive procedure stopped after 4,250, 3,750, and 4,000 gene-level randomizations, respectively, after satisfying the prespecified stability rule.

Table S9 reports up to 20 genes per module pair satisfying $q_{\text{within-pair}} < 0.07$. Genes were ranked primarily by decreasing observed MoBC score. Genes satisfying only the expanded threshold are explicitly identified so that the primary and borderline evidence tiers can be distinguished.

Table S9. Key-link gene prioritization at the selected settings using primary and expanded within-pair FDR thresholds. The primary count includes genes with $q_{\text{within-pair}} < 0.05$, whereas the expanded count includes all genes with $q_{\text{within-pair}} < 0.07$. Up to 20 genes meeting the expanded threshold are shown in decreasing order of observed MoBC score. Genes marked with † had $0.05 \le q_{\text{within-pair}} < 0.07$ and therefore met only the expanded screening threshold. Unmarked genes had $q_{\text{within-pair}} < 0.05$. Ties in observed MoBC score were resolved sequentially using lower $q_{\text{within-pair}}$, lower empirical p, higher standardized MoBC, and alphabetical gene symbol.

Network	Setting	Pair	Primary $q_{\text{within-pair}} < 0.05$	Expanded $q_{\text{within-pair}} < 0.07$	Ordered top 20 under the expanded threshold
BRCA-PPI	Walktrap 4	M2–M3	24	56	IL1B, CD44, TGFB1, TLR4†, APOE, DCN, SHC1, HPX, COL2A1, TLR2†, FGF23, A2M, EPHA2, PCOLCE, VTN†, MMP2†, LOX†, AREG, IL13†, IHH
BRCA-PPI	Infomap 0.75	M2–M7	48	63	TGFB1, CD44, COL2A1, DCN, FGF23, IL1B, SPARC, EPHA2, PCOLCE, HSPG2, SHC1, THBS1, FGF7, THY1, IHH, PDGFRB, WNT1, NAMPT, PTPRZ1, ENPP1†
CAC-PPI	Walktrap 3	M4–M8	36	38	Gnaq, Cacna1a, Agtr1a, Cacna1b, Agt, Gnai2, Gnai3, Cacna1s, Adk, Htr1f, Npy, Gnai1, Gna11, Cacna1f, Cacna1d, Nppa, Adcy9, Adcy5, Adcy3, Adcy1

8. ADDITIONAL COMPARATIVE KEY-LINK GENE ANALYSES

A. MoBC link genes between modules M1 and M2 in CAC

In the network of CAC, the top link genes between modules M_1 and M_2, were Il6 as the only link gene within shared JAK–STAT pathway (Fig. S21(a)). In particular, MoBC-Net showed that Il6 connected cytokines in M_1 to FoxO and JAK–STAT pathway genes in M_2 (Fig. S21(b)).

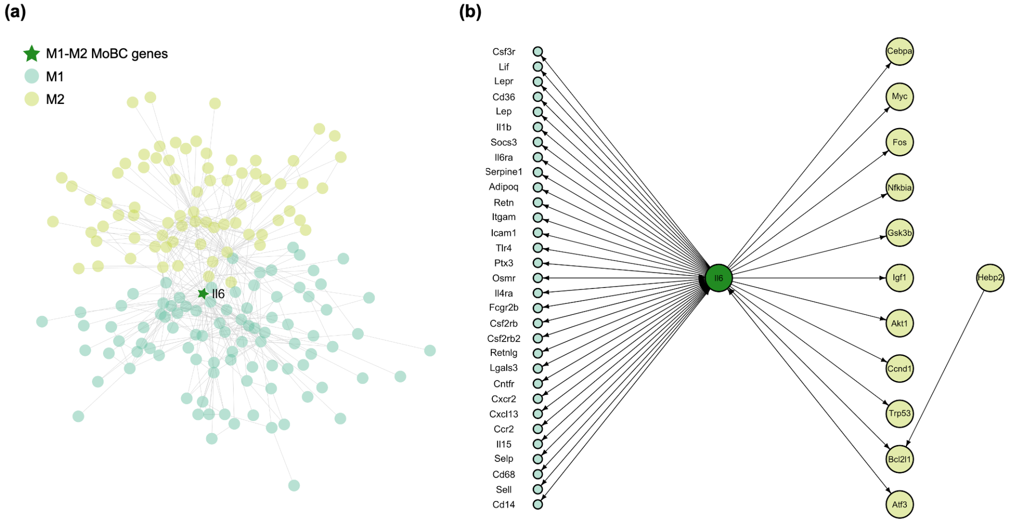

Fig. S21. Crosstalk module pair in the background network and the paths of a key link gene from CAC datasets. (a) The subnetwork shows interactions between CAC modules M_1 (light green) and M_2 (green). Top MoBC link genes involved in the JAK-STAT signaling pathway are marked with dark green stars. (b) The directed-path graph highlights Il6 (dark green) as the key MoBC link gene; arrows show the directed path between M_1 (light green) and M_2 (green) nodes via Il6.

B. Relationship of key link genes between MoBC, S2B, and FCS

We compared key link genes identified by MoBC, S2B, and FCS (Fig. S22). The ranks of MoBC-identified key link genes were generally consistent with their ranks obtained by S2B and FCS, indicating that the methods prioritized partially overlapping bridge genes.

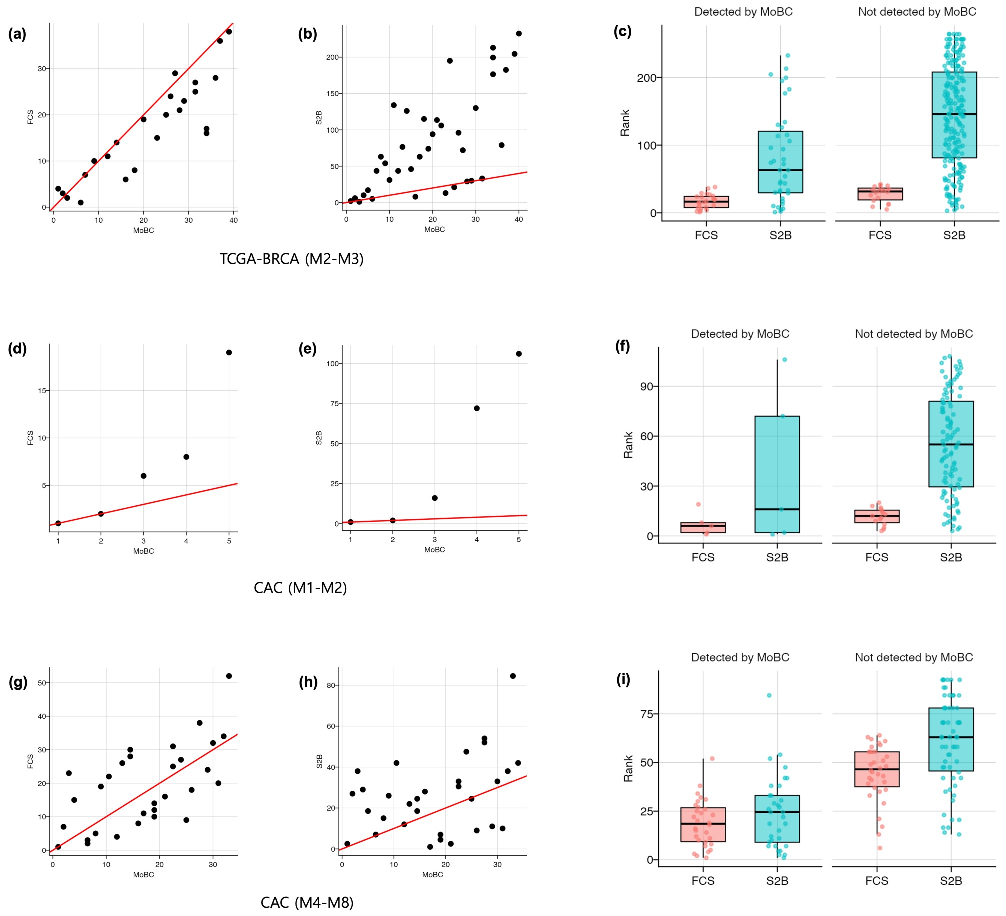

Fig. S22. Comparison of MoBC key link genes rankings obtained by MoBC, FCS, and S2B. Panels (a,d,g) show the ranks of MoBC-identified key link genes according to FCS, whereas panels (b,e,h) show their ranks according to S2B. Panels (c,f,i) compare the MoBC ranks of genes also identified by FCS or S2B with those not identified by MoBC. Results are shown for TCGA-BRCA M_2–M_3, CAC M_1–M_2, and CAC M_4–M_8 in panels (a–c), (d–f), and (g–i), respectively.

9. PROTEOMIC AND LITERATURE-BASED BIOLOGICAL SUPPORT

MoBC-Net link genes are selected from background-network topology and need not be DEGs. We therefore examined orthogonal protein-level and literature evidence for representative genes, including genes in the selected top-20 lists.

Protein abundance was drawn from DepMap proteomic 20Q2, a mass-spectrometry profile of Cancer Cell Line Encyclopedia cell lines [7]. EPHA2 occurs in both selected BRCA-PPI top-20 lists. Although it was not selected as a BRCA transcriptomic DEG, it showed subtype-dependent protein abundance and higher abundance in basal-like breast cancer cell lines (Figure S23). This agrees with reports linking EPHA2 to aggressive breast cancer and therapeutic relevance [13, 15, 17].

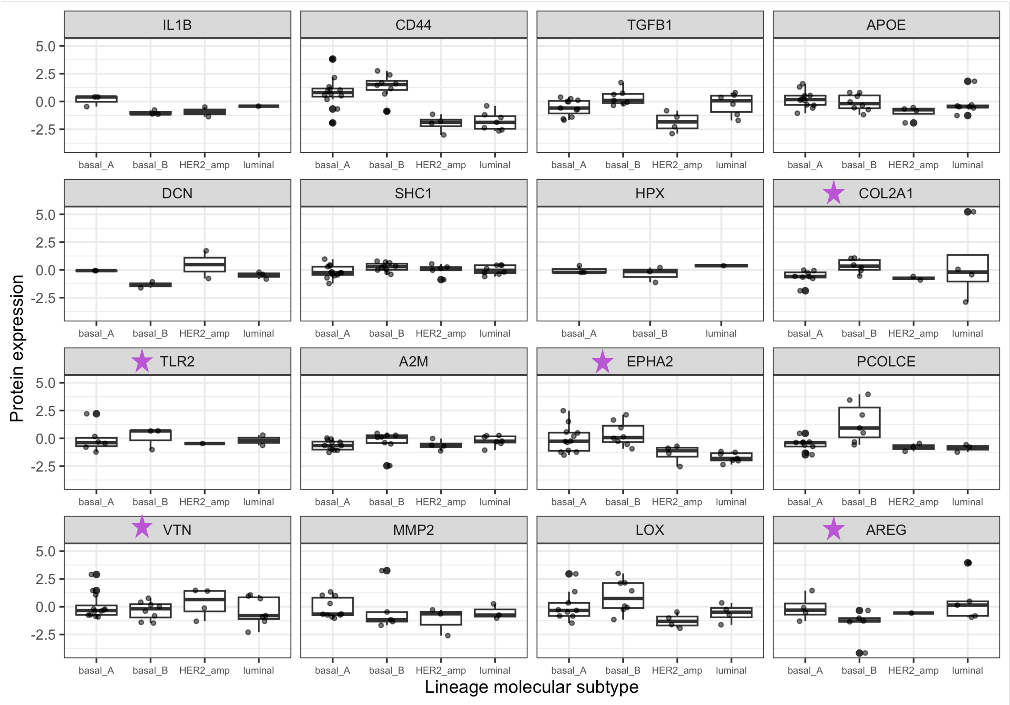

Fig. S23. External CCLE protein-level support for representative BRCA MoBC-Net link genes. Purple stars denote genes highlighted in the main manuscript.

GNAI2 occurs in the selected CAC-PPI Walktrap M4–M8 top-20 list. It encodes $G\alpha_i 2$, which promotes skeletal-muscle hypertrophy, myoblast differentiation, and regeneration [6]. Its placement between cAMP/GPCR-related and calcium/contractile modules is also compatible with evidence that impaired cAMP signalling contributes to mitochondrial dysfunction and muscle wasting in cancer cachexia [4]. These observations provide orthogonal plausibility, but they are not a formal external predictive-performance test.

REFERENCES

1. Vincent D. Blondel, Jean-Loup Guillaume, Renaud Lambiotte, and Etienne Lefebvre. Fast unfolding of communities in large networks. *Journal of Statistical Mechanics: Theory and Experiment*, 2008(10):P10008, 2008. doi: 10.1088/1742-5468/2008/10/P10008.
2. Marina L. García-Vaquero, Margarida Gama-Carvalho, Javier De las Rivas, and Francisco R. Pinto. Searching the overlap between network modules with specific betweenness (S2B) and its application to cross-disease analysis. *Scientific Reports*, 8:11555, 2018.
3. Emre Guney, Jörg Menche, Marc Vidal, and Albert-László Barabási. Network-based *in silico* drug efficacy screening. *Nature Communications*, 7:10331, 2016.
4. H. Ji and M. Rohm. Reawakening cAMP signalling in cancer cachexia. *Nat Metab*, 7:2388–2389, 2025.
5. Enrico Maiorino, Seung Han Baek, Feng Guo, Xiaobo Zhou, Parul H. Kothari, Edwin K. Silverman, Albert-László Barabási, Scott T. Weiss, and Benjamin A. Raby. Discovering the genes mediating the interactions between chronic respiratory diseases in the human interactome. *Nature Communications*, 11:811, 2020.
6. Giulia C. Minetti, Jerome N. Feige, Antonia Rosenstiel, Florian Bombard, Viktor Meier, Annick Werner, Frederic Bassilana, Andreas W. Sailer, Peter Kahle, Christian Lambert, David J. Glass, and Mara Fornaro. Gαi2 signaling promotes skeletal muscle hypertrophy, myoblast differentiation, and muscle regeneration. *Science signaling*, 4(201):ra80–, 2011.
7. David P. Nusinow, Jessica Szpyt, Mahmoud Ghandi, Christopher M. Rose, Edward R. McDonald, Marian Kalocsay, Judit Jane-Valbuena, Ellen Gelfand, Devin K. Schweppe, Mark Jedrychowski, Javad Golji, Dale A. Porter, Tomas Rejtar, Y. Karen Wang, Gregory V. Kryukov, Frank Stegmeier, Brian K. Erickson, Levi A. Garraway, William R. Sellers, and Steven P. Gygi.

Quantitative proteomics of the cancer cell line encyclopedia. *Cell*, 180(2):387–402.e16, 2020. doi: 10.1016/j.cell.2019.12.023.

8. Catia Pesquita, Daniel Faria, Hugo Bastos, Antonio E. N. Ferreira, Andre O. Falcao, and Francisco M. Couto. Metrics for GO based protein semantic similarity: A systematic evaluation. *BMC Bioinformatics*, 9, 2008. doi: 10.1186/1471-2105-9-S5-S4.
9. Pascal Pons and Matthieu Latapy. Computing communities in large networks using random walks (long version), 2005.
10. Philip Resnik. Using information content to evaluate semantic similarity in a taxonomy. In *Proceedings of the 14th International Joint Conference on Artificial Intelligence*, pages 448–453, 1995.
11. Martin Rosvall and Carl T. Bergstrom. Maps of random walks on complex networks reveal community structure. *Proceedings of the National Academy of Sciences*, 105(4):1118–1123, 2008. doi: 10.1073/pnas.0706851105.
12. Vincent A. Traag, Ludo Waltman, and Nees Jan van Eck. From louvain to leiden: Guaranteeing well-connected communities. *Scientific Reports*, 9(1):5233, 2019. doi: 10.1038/s41598-019-41695-z.
13. David B Vaught, Alyssa R Merkel, Conor C Lynch, James Edwards, Mohammed Noor Tantawy, Timothy Hilliard, Shan Wang, Todd Peterson, Rachelle W Johnson, Julie A Sterling, and Dana Brantley Sieders. EphA2 is a clinically relevant target for breast cancer bone metastatic disease. *JBMR Plus*, 5(4):e10465, 01 2021.
14. J. Z. Wang, Z. Du, R. Payattakool, P. S. Yu, and C. F. Chen. A new method to measure the semantic similarity of go terms. *Bioinformatics*, 23(10):1274–1281, 2007.
15. Ta Xiao, Yuhang Xiao, Wenxiang Wang, Yan Yan Tang, Zhiqiang Xiao, and Min Su. Targeting EphA2 in cancer. *Journal of Hematology & Oncology*, 13(114), 2020.
16. Guangchuang Yu, Fei Li, Yide Qin, Xiaochen Bo, Yibo Wu, and Shengqi Wang. GOSemSim: an R package for measuring semantic similarity among GO terms and gene products. *Bioinformatics*, 26(7):976–978, 2010.
17. Ping Zhao, Dewei Jiang, Yunchao Huang, and Ceshi Chen. EphA2: A promising therapeutic target in breast cancer. *Journal of Genetics and Genomics*, 48(4):261–267, 2021.
